# On the Morphological Description of Tracheal and Esophageal Displacement and Its Phylogenetic Distribution in Avialae

**DOI:** 10.1371/journal.pone.0163348

**Published:** 2016-09-20

**Authors:** Jeremy J. Klingler

**Affiliations:** Department of Biological Sciences, Northern Illinois University, DeKalb, Illinois, United States of America; New York Institute of Technology College of Osteopathic Medicine, UNITED STATES

## Abstract

This research examines the evolution and phylogenetic distribution of a peculiar and often overlooked character seen in birds, herein called tracheal and esophageal displacement. Tracheal and esophageal displacement refers to an asymmetrically situated trachea and/or esophagus along the length of the neck. This contrasts with what would be perceived as the “normal” (midsagittal) placement of these organs, wherein the two organs are situated along the ventral midline of the neck with no deviation. A total of forty-two bird species were examined (thirty-six of which came from dissections whereas six came from comments from previous literature or personal observations), as well as turtles, lizards, crocodylians, and mammals. This study found that essentially all birds have a laterally displaced trachea and/or esophagus. Lizards and mammals were seen to have normal, midsagittally placed tracheae and esophagi. Crocodylians were interesting in that alligators were defined by a normally situated trachea and esophagus whereas some crocodiles were characterized by displacement. In birds, the displacement may occur gradually along the neck, or it may happen immediately upon exiting the oropharynx. Displacement of these organs in birds is the result of a heavily modified neck wherein muscles that restrict mobility of the trachea and esophagus in lizards, alligators, and mammals (e.g., *m*. *episternocleidomastoideus*, *m*. *omohyoideus*, and *m*. *sternohyoideus*) no longer substantially restrict positions of the trachea and esophagus in birds. Rather, these muscles are modified in ways which may assist with making tracheal movements. The implications of this study may provide interesting insights for future comparisons in extinct taxa.

## Introduction

Despite a wealth of books and studies devoted to avian anatomy, very little attention has been paid to the relative positions of the cervical viscera (which includes the trachea, the esophagus, the jugular vein, the carotid artery, the vagus and sympathetic nerves, and other tissues) in birds or other vertebrates. Owing to a great understanding of mammalian morphology, it might seem intuitive to think that the position of all other vertebrate cervical viscera, in particular the tracheae and esophagi, would be much as in humans, with the trachea and esophagus both being directly midsagittal in the neck. However, the relative positions of these anatomical features have really only been examined sparsely or noted in detail in comparative studies. These mentions are only brief, vague, and made in passing. Although it may seem unimportant or maybe even obvious, an otherwise fascinating aspect regarding the esophagus and trachea has therefore been overlooked. The esophagus and trachea are actually often greatly laterally displaced in birds. That is, rather than a midsagittal placement relative to the body of the neck (vertebrae and their surrounding muscles) for the trachea and the esophagus, these structures may lie lateral to the body of the neck. In contrast to the trachea and esophagus, the rest of the cervical viscera (i.e., the external carotids, the jugular veins, and vagus nerve) occupy their respective sides (e.g., the left jugular vein remains positioned on the left side of the neck whereas the right jugular vein remains on the right side of the neck). Simple dissection of the necks of vertebrates easily reveals displaced cervical viscera, but aside from anecdotal descriptions (e.g., [1–10]), no study has rigorously explained the system and its variation in birds and reptiles. Hereafter, this morphological occurrence is named tracheal and esophageal displacement, or TED.

The following questions will be addressed here. First, what unique anatomical features or structural modifications allow for this arrangement to exist in birds? Second, what is the phylogenetic occurrence of displacement both in extant aves or other amniotes, such as non-avian outgroups (e.g., turtles, lepidosaurs, and non-avian archosaurs)? Third, what is the function of having displaced cervical viscera? Future papers will discuss whether extinct fossil forms displayed displacement.

The aim of this study is to provide the first detailed anatomical description of tracheal and esophageal displacement in the context of explaining what allows it as well as understanding its phylogenetic occurrence. To understand tracheal and esophageal displacement, a working definition is generated to recognize it. Tracheal and esophageal displacement is defined here as any of a number of asymmetrical conditions seen in vertebrates wherein either the trachea, the esophagus, or both are laterally offset from the midline of the neck for an appreciable length of the neck and/or thorax. As will be examined later, several different conditions of lateral displacement exist. These lateralized conformations contrast with what might be thought of as a “normal” condition where both the trachea and the esophagus lie in the ventral midline of the neck.

It is hypothesized here that lateral displacement of the cervical viscera evolved in birds to function as an ever increasingly efficient bypass system to allow the trachea to remain a short, straight, and patent tube able to keep up with the demands of a more mobile and flexible neck. A more loosely attached trachea and esophagus would be beneficial for those birds with highly dynamic neck movements.

This study’s scope encompasses functional morphology, comparative anatomy, and phylogenetics to infer paleobiological interpretations in future works.

## Methods

A general comparative anatomical survey of neontological specimens was conducted. Morphological data were collected by dissecting the necks of a wide range of extant organisms.

### Dissections

Data were collected from the dissection of a number of amniotes. Photographs of specimens were taken using a Canon^®^ EOS 5D Mark II camera and a Samsung^®^ ST150F camera (16.2MP Smart WiFi digital camera with 5x optical zoom and 3.0” LCD screen). A myriad of extant species were dissected to develop a substantial phylogenetic comparison. Species bought through Carolina Biological Supply Company (Burlington, NC) included: *Pseudemys concinna*, *Anolis carolinensis*, and *Columba livia* preserved in Carosafe^®^. Four bird specimens came from the Northern Illinois University Department of Biological Sciences. The majority of birds were salvaged in accordance with the Illinois Department of Natural Resources Scientific Collecting Salvage Permit (Permit Number: NH14.5773). These specimens were fresh, frozen, and then thawed at the time of dissection. Two juvenile female *Alligator mississippiensis* specimens were provided by the Rockefeller Wildlife Refuge with a Louisiana Department of Wildlife and Fisheries Special Alligator Permit. A specimen of *Bradypus tridactylus* preserved in alcohol was examined which came from Northern Illinois University. All specimens personally dissected are listed in Table 1 below. Remarks made of other taxa came from the literature and personal observations.

**Table 1:** Specimens Dissected.

Species	Number of Individuals
**Chelonia**	
*Pseudeyms concinna*	2
**Lacertilia**	
*Anolis carolinensis*	6
**Crocodylia**	
*Alligator mississippiensis*	2
**Aves**	
*Branta canadensis*	2
*Aix sponsa*	2
*Anas platyrhynchos*	2
*Meleagris gallopavo*	2
*Columba livia*	7
*Zenaida macroura*	1
*Fulica americana*	2
*Rallus limicola*	1
*Coccyzus americanus*	1
*Phalacrocorax auritus*	4
*Ardea herodias*	2
*Larus delawarensis*	3
*Buteo jamaicensis*	2
*Buteo platypterus*	1
*Accipiter cooperii*	1
*Bubo virginianus*	2
*Strix varia*	1
*Megascops asio*	1
*Megaceryle alcyon*	1
*Sphyrapicus varius*	2
*Falco sparverius*	1
*Hirundo rustica*	1
*Certhia americana*	1
*Turdus migratorius*	2
*Catharus guttatus*	1
*Bombycilla cedrorum*	1
*Seiurus aurocapilla*	3
*Wilsonia canadensis*	1
*Geothlypis trichas*	1
*Junco hyemalis*	1
*Pheucticus ludovicianus*	1
*Cardinalis cardinalis*	2
*Quiscalus quiscula*	1
*Agelaius phoenicius*	1
*Carpodacus mexicanus*	1
*Spinus tristis*	2

### Radiographs

Radiographs of many specimens were taken prior to dissection to ensure accurate interpretation of tracheal and esophageal placement before any potential unintended manipulation occurred as a result of the handling of the specimen and during its dissection. Radiographs were taken using a GE MOBILE 100–15 X-ray unit.

### Phylogenetics

An ancestral state reconstruction with stochastic character mapping [11] and Pagel’s lambda [12] were calculated using the statistical program R in order to understand the evolution of tracheal displacement in birds. R packages used included ‘APE’ [13], ‘geiger’ [14], and ‘phytools’ [15]. Esophageal displacement did not vary enough to perform a meaningful analysis. The phylogenetic trees made followed the Hackett et al. [16] all species backbone from birdtree.org. Further species with conditions known from personal observations and the primary literature were also added.

### Comparison of the lengths of the neck and tracheae

The length of the neck compared to the length of the trachea along the neck was measured in a few birds. These lengths were measured from photographs of specimens where the lengths of the ventral curves of the necks (arc lengths) were measured by simple approximation by multiple hypotenuses. Lengths of tracheae were calculated as simple linear segments as they course more or less straight. The lengths of the tracheae and necks were measured while in a “neutral” state, with the neck in a more or less S-shape, and in a stretched state, where the neck was stretched forward reducing the S-shape, straightening the neck. This comparison reveals how much distance the trachea must cover when the neck is in an extended and a normal posture. Esophageal lengths along the neck are assumed to be equal to tracheal lengths.

## Results

### Phylogenetic distribution of displacement

This section discusses the details of the course and positions of the cervical viscera, namely the trachea and esophagus, and whether certain vertebrates exhibit tracheal and esophageal displacement. Examples of exceptionally preserved fossil taxa will be discussed in a future paper.

### Turtles, snakes, and lizards

#### Order Chelonia; Order Squamata

**Fig 1:**
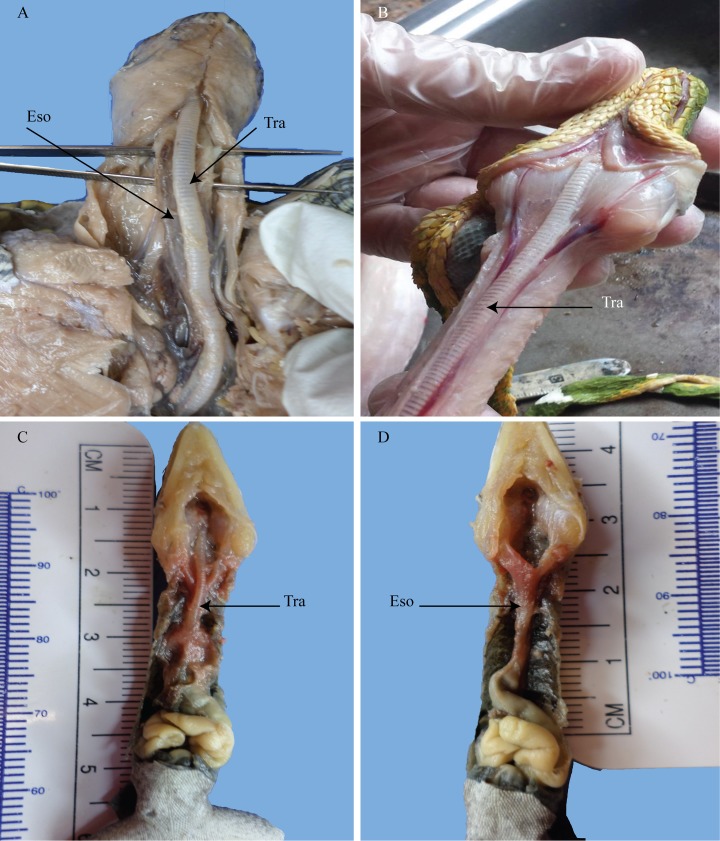
Ventral views of *Pseudemys*, *Corallus*, and *Anolis*. (A) the turtle trachea and esophagus are seen to pass straight. (B) the emerald tree boa’s trachea and esophagus are seen to stay in the midline. (C) the anolis trachea’s course is straight. (D) the anolis esophagus passes midsagittally. Tra = trachea; Eso = esophagus. Emerald tree boa image courtesy of R. DePalma.

The eastern river cooter (*Pseudemys concinna*) showed a normal, midsagittal, positioning of the esophagus and trachea (Fig 1A). Both elements are tightly surrounded by thick hyoid and neck musculature. Although this turtle exhibits a normal midsagittal route for the trachea and esophagus along the length of the neck, once inside the thorax, the organs exhibit a slight, insubstantial, though noticeable left leaning. The stomach, for instance, is positioned on the left side of the body. Therefore, the esophagus must detour significantly to the left to reach it. Interestingly, near the point where the trachea branches into the bronchi, it rotates to lie on its side. Thus, where the bronchi enter the lungs they too lie on their sides. The esophagus is consistently flat along its length, whereas the trachea remains subcircular.

Interestingly, even though this particular species observed did not demonstrate displacement, some turtles do. Zehtabvar et al. [17] observed in the European pond turtle (*Emys orbicularis*) that the trachea diverged off to the left side of the neck as early as the 3^rd^ cervical vertebra. Further, Valente et al. [18] reported that the trachea diverts off to the right side of the neck in the loggerhead turtle (*Caretta caretta*). Personal observations of CT scans of a mata mata (*Chelus fimbriata*) also revealed a left laterally displaced trachea.

The emerald tree boa (*Corallus caninus*) has a straight trachea and esophagus (Fig 1B), although it does not appear to be wholly restricted on the sides. As this specimen was not personally dissected, nor were other snake specimens, it is not possible to say much with regard to snake tracheal and esophageal features. Regrettably, I had few snakes to test experimentally, but it remains convincing to think that those snakes that consume large boli should be able to slide their tracheae to the side to prevent crushing of the rings. In fact, this is probably the case for a number of snakes. Wallach [19] noted that in many snakes the trachea may lie right ventrolateral or right lateral to the esophagus. Hence, at least some snakes do exhibit displacement.

The Carolina anolis (*Anolis carolinensis*) conforms to a normal tracheal and esophageal position with a typical midsagittal placement (Fig 1C and 1D). The undistended esophagus is at its broadest and flattest shape in the most immediate cranial region. Shortly thereafter, the esophagus becomes circular as it approaches the stomach. Observations of CT scans of other lizards including iguanas and monitor lizards showed normal, midsagittal, positions.

### Crocodylians

#### Superorder Archosauria; Order Crocodylia

Two juvenile American alligators (*Alligator mississippiensis*) were dissected. The specimens were 102.9 cm and 91.4 cm in total length. The total tracheal length from the posterior end of the larynx to the carina of the 102.87 cm specimen was 14 cm. It was about nearly equal in lateral diameter throughout its length (9.5–9.2 mm), except for the more posterior region, and especially in the most caudal region, as it approached the carina. At this point, it became a millimeter smaller (8.5 mm). The trachea is somewhat flattened, making a more ovoid ring with a dorsoventral diameter ranging from about 5 mm cranially to 4 mm caudally. Once inside the thorax, the trachea became smaller and more circular. The alligator trachea exhibits between 50–60 cartilaginous rings [20]. There were 60 rings in the 102.9 cm long individual. The number of rings does not increase with age [21]. Crocodylians exhibit a complete trachea. However, a small number of those rings may in fact be open dorsally, making them incomplete C-shaped rings, which widen as the trachea approaches the larynx [21]. The bronchi may also be open in young individuals; however, they close quickly [20].

The esophagus of the alligator contains many longitudinal folds which increase the range of distension for the accommodation of large boli. In crocodylians the esophagus is separated from the oral cavity and the entrance to the trachea by a transverse fold. This structure projects from the floor of the mouth and joins a shorter fold descending from the cranial floor [22].

**Fig 2:**
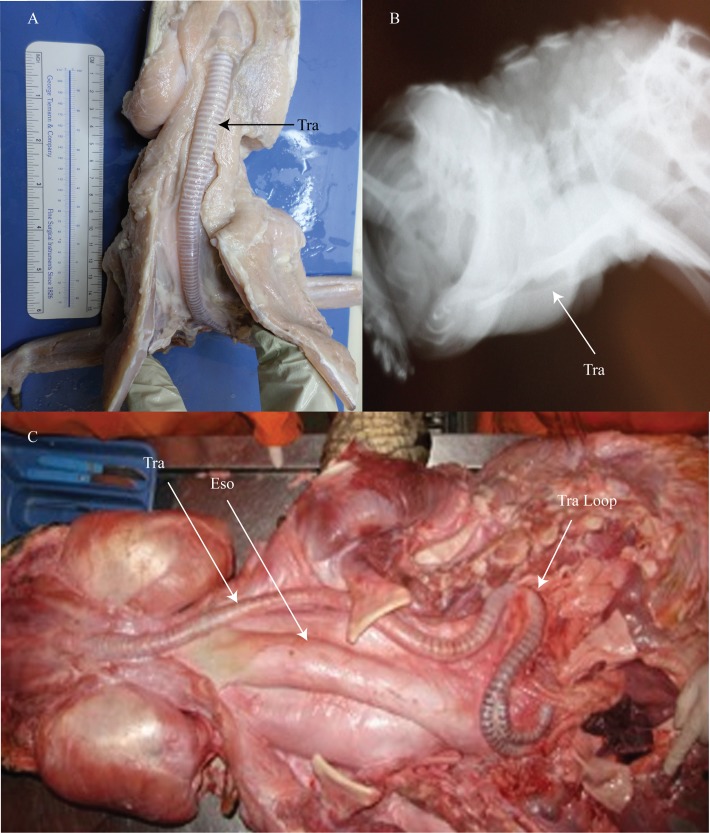
*Alligator mississippiensis* and *Crocodylus niloticus*. (A) ventral view of *A*. *mississippiensis* trachea. (B) radiograph of alligator trachea. (C) ventral view of the Nile crocodile’s trachea and esophageal course. Tra = trachea; Eso = esophagus; Tra loop = tracheal loop. Nile crocodile image courtesy of J. Reidenberg.

The alligators show the typical midsagittal placement of both the trachea and the esophagus (Fig 2A and 2B). Once inside the thorax, though, the trachea became slightly dorsally deflected. It was observed that the trachea was capable of some mobility and thus potentially could be lateral at times. For instance, the organs could be lateralized in instances where large boli might push the trachea ventrolaterally. In fact, sectioning the neck of an alligator showed this. In the anterior region, the trachea is firmly held in the midline. Even after having expanded the esophagus a considerable degree to simulate the presence of a large bolus, the trachea remained in the ventral midline firmly attached to infrahyoid muscles. The trachea would not move farther to the side without bringing those muscles with it. As in the anterior region, the trachea was initially in the ventral midline of the posterior region. Contrary to the anterior region, though, in the posterior region, when the esophagus was expanded, the trachea easily and readily slid somewhat laterally. The reason for the asymmetrical slide in this region is due to the presence of the thin, flat, anteriorly projecting episternum. The episternum’s presence creates a necessity for lateral sliding as a largely distended esophagus might otherwise crush a trachea that was fixed in the midline position. If such is the case in a living specimen, then lateralization would only be brief and circumstantial. Ultimately, though, displacement was not observed as the primary condition, and it probably is not likely in *A*. *mississippiensis* except perhaps momentarily when substantially sized boli pass along the neck.

Although no crocodilid specimens were available for dissection, photographs, diagrams, and literature reviews of the Nile crocodile (*Crocodylus niloticus*) provided very useful insights into crocodilid anatomy. Alligatorid and crocodilid crocodylians differ in three important ways. First and foremost is the presence of tracheal elongation and looping. Tracheal elongation and looping occurs when the trachea is excessively long, longer than the necessary length to reach from the oropharynx to the lungs. Reese [20] observed that many crocodylians exhibit looping, and that some species exhibit this looping before hatching while others not until well after hatching. Elongation and looping does not occur in *A*. *mississippiensis* [20]. This was confirmed from specimens in this study. Schachner et al. [23] noted that smaller individuals of the Nile crocodile lacked looping, while older and larger specimens displayed notable and significant tracheal elongation and looping. The second important distinction is that crocodilids, unlike alligatorids, exhibit tracheal displacement. Schachner et al. [23] showed that in smaller crocodilid crocodylian specimens the trachea and the esophagus were situated in the ventral midline of the neck. Interestingly, though, [23] further noted that asymmetry of the trachea exists in larger crocodylians as the trachea actually passes along the side of the esophagus making marked hairpin turns before entering the lungs (Fig 2C). Reese [20] also suggested that there was a lateral bend in the trachea of gavials (Family: Gavialidae). The displaced trachea in the Nile crocodile does not appear to exhibit any rotation onto the side, but rather remains situated so that the dorsal side of the trachea faces dorsally throughout. Though this is not confirmed via direct observations. The last major distinction between *A*. *mississippiensis* and *C*. *niloticus* was that aside from an elongated trachea, the primary extrapulmonary bronchi were also elongated, being drawn out into an S-shaped curve laterally, caudally, and dorsally [23]. The elongation of the bronchi also positions the carina of the trachea more proximally [23].

### Birds

#### Class Avialae

**Fig 3:**
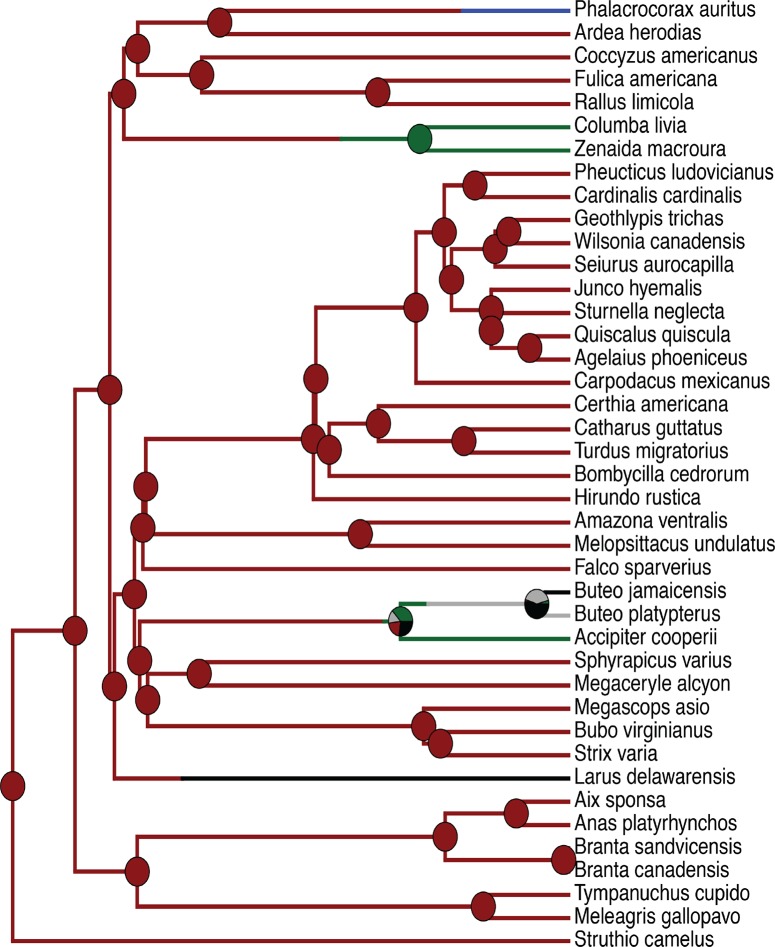
Ancestral state reconstruction phylogeny of tracheal displacement. For ease of understanding tracheal placements in bird taxa. Esophageal placement has, essentially, almost no variability and so is not plotted. Red = right lateral; green = left lateral; black = right lateral and left lateral; blue = right lateral and midsagittal; gray = midsagittal.

Fig 3 below illustrates an ancestral state reconstruction of the results of looking at the tracheal positions of numerous bird species. Pagel’s lambda was calculated for the trait ‘tracheal displacement’ from a random sample of 100 trees from the posterior distribution of trees recovered in the analysis by Hackett et al. [16], and resulted in a median lambda value of 0.900652 indicating a high phylogenetic signal for tracheal displacement.

Due to the overwhelming similarities between different species of birds, a cursory treatment of what is seen in various bird orders is given here.

#### Superorder Palaeognathae; Order Struthioniformes

Tivane [6] noted that in ostriches (*Struthio camelus*), the cranial portion of the esophagus lies in the midline and dorsal to the trachea to which it is attached along its ventrum by connective tissue. More caudally, the esophagus is situated laterally to the right of the midline [6]. Various other researchers [7–9] observed the lateralization of the esophagus in the ostrich, and that it lies on the right, lateral and dorsal to the trachea. Furthermore, Huchzermeyer [24] and Bezuidenhout [9] noted that in ostriches at the level of the sixth cervical rib, the esophagus expands into the proventriculus. The esophagus is mobile and expandable, and ratites swallow their food whole [25]. The crop is absent in all ratites [26].

The trachea has complete cartilaginous rings and makes a tube 114.3 cm in length [1]. Owing to its mobile nature, the trachea may certainly become lateralized in the posterior region. Observations of the necks of captive, living individuals also indicates a laterally displaced trachea which may not become situated laterally on the right until the mid to caudal regions. This has been observed in living individuals due to their large size and the lack of feathers covering the neck.

#### Superorder Neognathae; Order Galliformes; Order Anseriformes; Order Columbiformes

Personal observations of a juvenile prairie chicken, *Tympanuchus cupido*, revealed a tracheal and esophageal placement in which both organs lie together on the right side of the neck. The presence of a crop did not cause a divergence of the trachea away from the esophagus, but rather the two passed together. Beginning around the end of the midsection and the start of the posterior region of the neck, the trachea and esophagus shift to the right side of the neck. The turkey (*Meleagris gallopavo*) exhibited tracheae and esophagi that passed to the right side of the neck gradually, not reaching the side of the neck until the posterior region.

**Fig 4:**
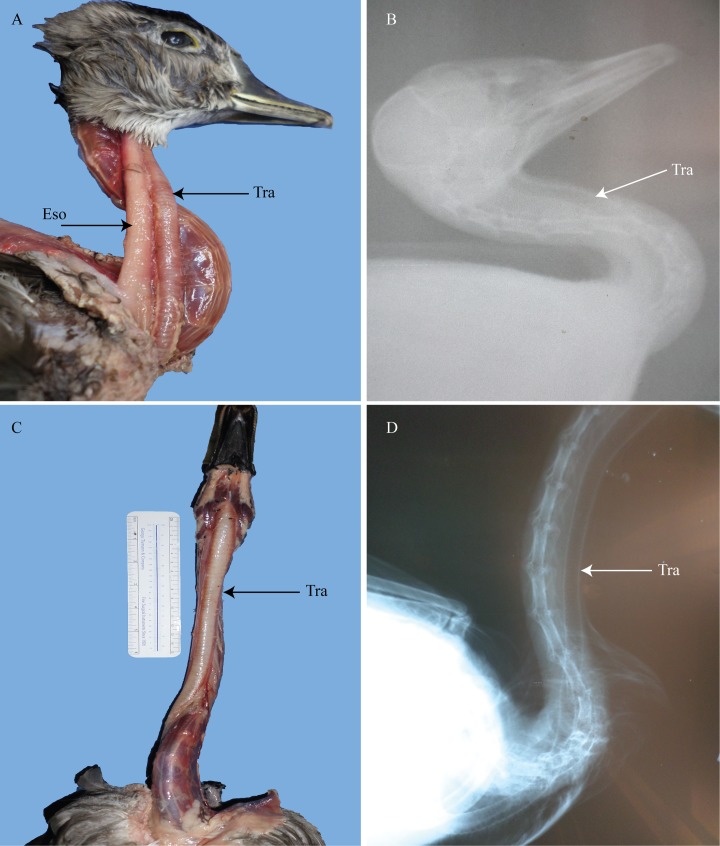
Duck and goose trachea and esophagus. (A) right lateral view of the neck of *Aix sponsa* showing displacement of the organs occurring quickly after exiting the oropharynx. (B) radiograph of *Aix sponsa*. (C) ventral view of the neck of *Branta canadensis* showing a gradual displacement of the organs so that they are lateralized only in the posterior region of the neck. (D) radiograph of lateral view of *Branta canadensis*. Tra = trachea; Eso = esophagus.

The wood duck (*Aix sponsa*) and the mallard (*Anas platyrhynchos*) both exhibited displacement of the trachea and esophagus onto the right side of the neck. Unlike geese and turkeys, the ducks show a more immediate displacement where the organs become situated laterally almost immediately upon exiting the oropharynx (Fig 4A and 4B). Upon approaching the end of the cranial region, the two organs rotated so that the dorsal sides faced medially. Additionally, due to the curvature of the neck, the two may lie dorsolaterally in the caudal region of the neck. Both male and females showed the same arrangements with the trachea and esophagus coursing along the ventral midline in the upper neck while lateralizing and rotating starting somewhat before the middle of the neck.

In the Canada goose (*Branta canadensis*) and the Hawaiian goose (*Branta sandvicensis*; as described by Humphrey [27]), the trachea and the esophagus are situated along the midline of the neck for most of its length. Only in the posterior region did the trachea and esophagus become situated on the right side (Fig 4C and 4D). Much like the ostrich and turkey, these geese display a gradual displacement. The trachea of the Canada goose was further seen to dip well below the cervical column in the cervicodorsal region at a 50° angle where it makes contact with the hypocleidum of the furcula and enters the interclavicular air sac. It then curves up the *m*. *pectoralis secundus* at a 40° angle, entering the thoracic cavity back from the midline. This ventral dipping creates a slightly elongated trachea. For a review of which species of birds exhibit an elongated trachea see Fitch [28]. While Fitch [28] did not discuss or list *B*. *canadensis* as a species which exhibits elongation, this study does. In the posterior region of the neck, the trachea rotates, to lie slightly on its side as it courses from a lateral position closer to a mid-ventral position. The esophagus, however, remains tightly appressed to the cervical column throughout its length.

**Fig 5:**
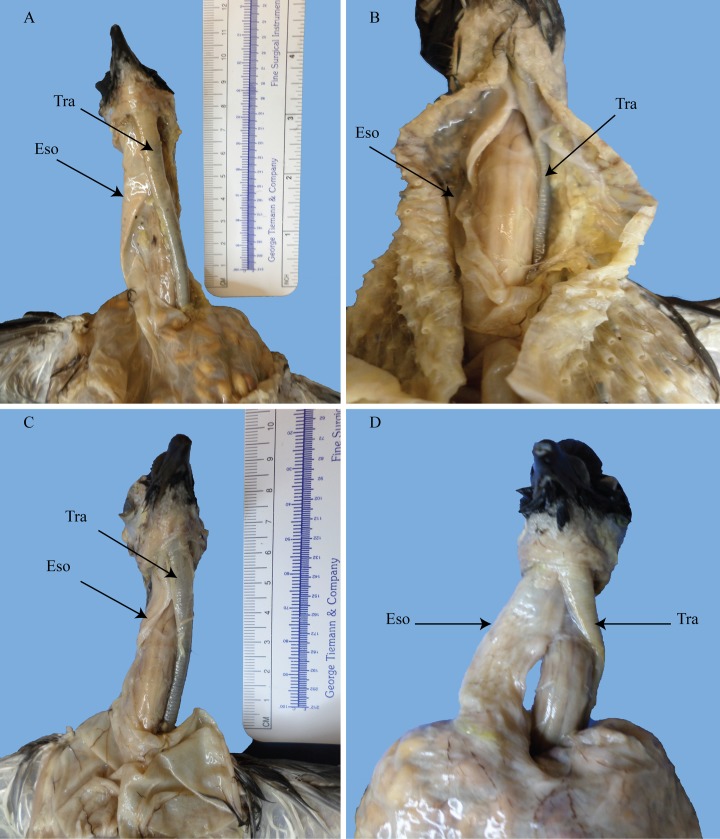
Pigeons showing individual variation of the degree and immediacy of displacement. A and C show a more gradual displacement of the trachea while B and D show a more immediate displacement of the trachea. D also shows a strongly dorsolateral placement of the trachea having been pushed dorsally due to a filled crop. Tra = trachea; Eso = esophagus.

In contrast to what is observed in, for example, the ducks, where both the trachea and esophagus lay together on the same side, the rock pigeon (*Columba livia*) and the mourning dove (*Zenaida macroura*) achieve tracheal and esophageal displacement in a unique way involving the two organs diverging so that each organ is on a different side of the neck. The undistended esophagus lies flat along the right lateral/ventrolateral side of the neck. Due to a specialized and large, bilobed crop, the esophagus in the pigeon was considerably shortened. Also affected by the crop was the trachea, which was deflected dorsally in the more posterior region of the neck as it approached the thoracic inlet. When the crop is full, the trachea may be greatly deflected dorsally as the crop covers the thoracic inlet. The trachea, in contrast to the esophagus, lies along the left lateral side of the neck. Even within species, noticeable variation of tracheal and esophageal displacement exists in these birds to varying degrees. In some, the trachea and esophagus were only slightly displaced while in others they would be greatly displaced (Fig 5). The two organs lie together midsagittally only at the cranialmost segment of the neck, particularly right as they exit the oropharyngeal region. The two organs then diverge to the side of the neck either immediately thereafter or gradually.

#### Order Gruiformes; Order Cuculiformes; Order Suliformes; Order Pelecaniformes; Order Charadriiformes

Both the esophagus and trachea in the American coot (*Fulica americana*) exhibit a midsagittal position in the anterior and mid-cervical regions, only diverging to the right side of the neck at the posterior cervicals. The Virginia rail’s (*Rallus limicola*) trachea and esophagus proceeded toward a right lateral placement immediately upon leaving the oropharynx. At the middle region of the neck, the trachea rotates onto its side. Both the trachea and esophagus are considerably dorsolaterally placed. The yellow-billed cuckoo (*Coccyzus americanus*) demonstrated right lateral tracheal and esophageal displacement occurring quickly from the oropharynx. The trachea did not exhibit any rotational component.

**Fig 6:**
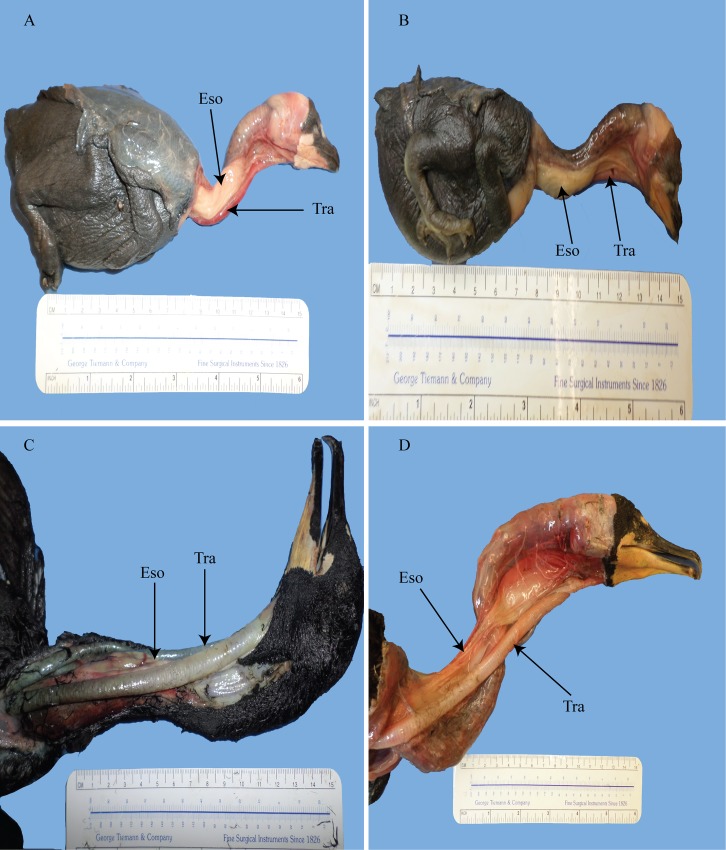
The course of the trachea and esophagus in *P*. *auritus*. (A and B) lateral view of hatchling *P*. *auritus*. (C) ventral view of juvenile *P*. *auritus* showing a midsagittal placement of the trachea. (D) lateral view of adult *P*. *auritus*. Tra = trachea; Eso = esophagus.

**Fig 7:**
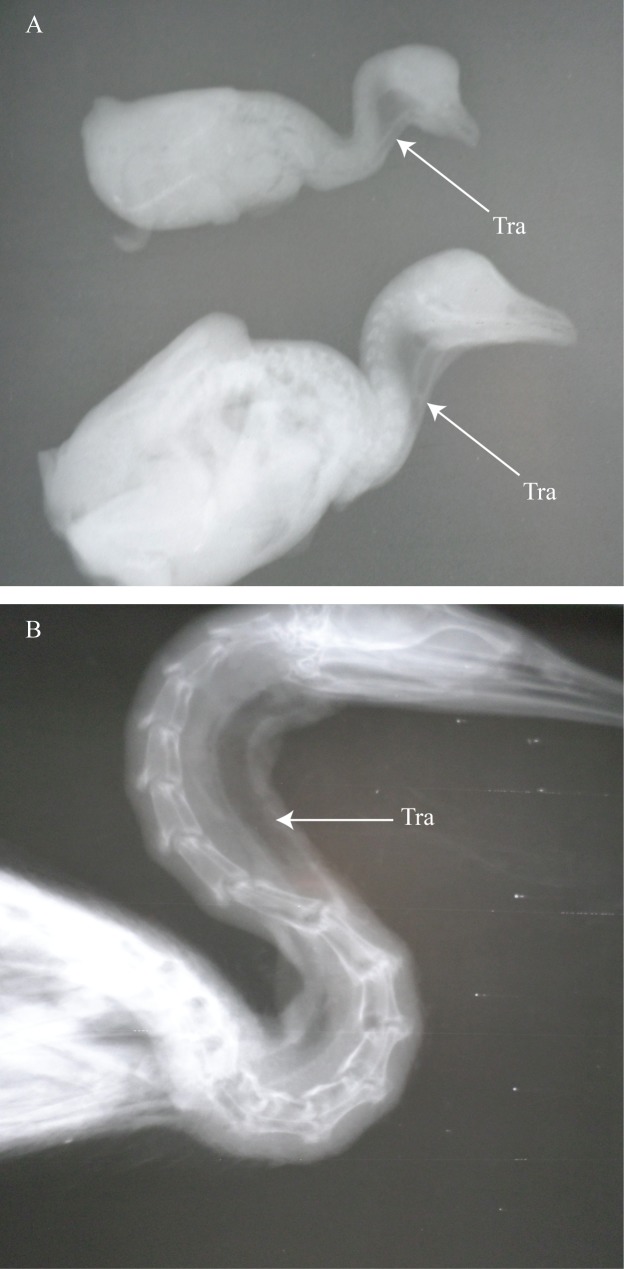
*Phalacrocorax auritus* radiographs. (A) hatchling double-crested cormorants. (B) adult. Tra = trachea.

An ontogenetic sampling of the double-crested cormorant (*Phalacrocorax auritus*) was available. The sample included two hatchlings, one juvenile (< 1 year old based on plumage), and one adult. The esophagus in both hatchlings was broad and lay well right lateral to the cervical column. In the smallest hatchling, the trachea was only slightly right lateral to the cervical column and only in the most posterior section (Figs 6A and 7A). In the larger hatchling, the trachea was displaced laterally throughout its length as was the esophagus (Figs 6B and 7A). The juvenile was odd in that the esophagus was somewhat right lateral whereas the trachea passed along the ventral midline (Fig 6C). The adult’s trachea and esophagus passed right laterally across the neck (Figs 6D and 7B).

Overall, the double-crested cormorant revealed that even in recent hatchlings, tracheal and esophageal displacement is already present. Both hatchlings showed tracheal and esophageal displacement to varying degrees. The juvenile’s “normal” placement of the trachea is odd as both infants and adults display lateralization. The midsagittal placement of the trachea is likely the result of it having such freedom and mobility to be able to be moved to the midline.

**Fig 8:**
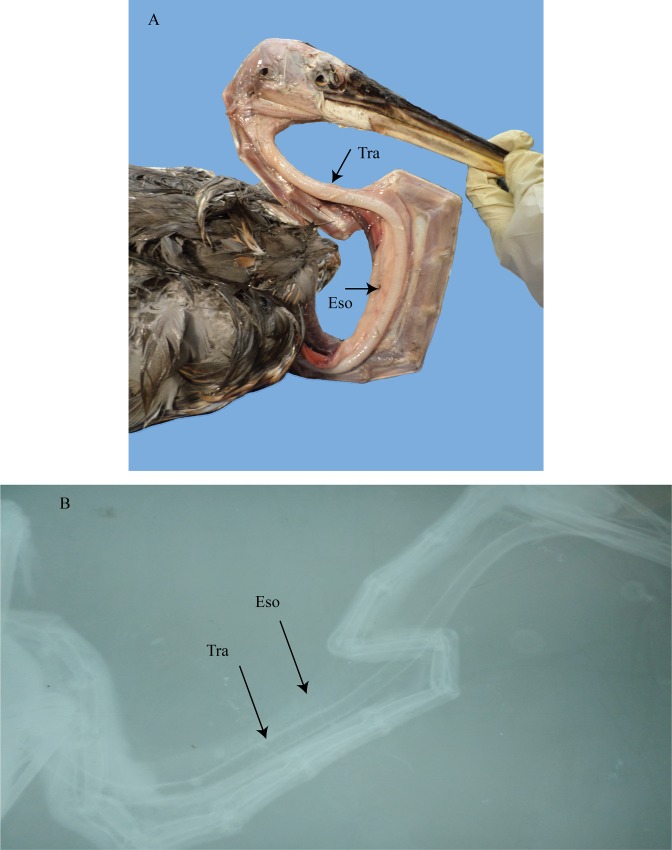
*Ardea herodias*. (A) dissection. (B) radiograph. Tra = trachea; Eso = esophagus.

The great blue heron (*Ardea herodias*) has a trachea and esophagus that travels 12 cm along the ventral midline of the neck until the fifth cervical vertebra where they pass right laterally across it to become situated well dorsal to the vertebral column (Fig 8). By the twelfth cervical vertebra they cut back across the cervical column to become positioned at the ventral midline once more to enter the thorax. As the trachea passes across the fifth cervical it also rotates onto its side. Overall, the trachea is circular, especially in the mid and posterior regions.

**Fig 9:**
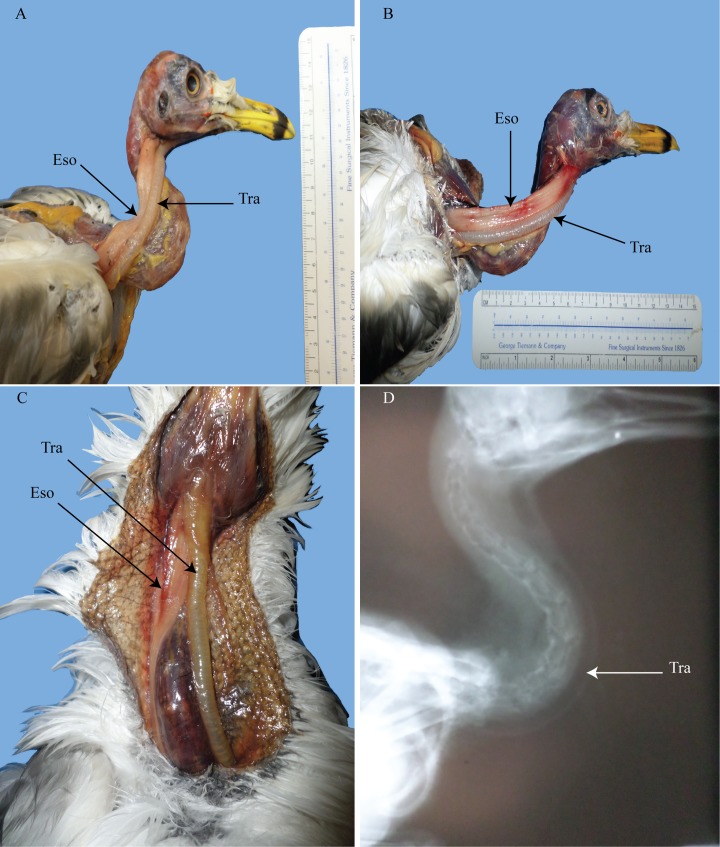
*Larus delawarensis*. (A) lateral view of ring-billed gull. (B) lateral view of ring-billed gull. (C) ventral view of ring-billed gull showing the trachea on the left side of the neck. (D) radiograph of the gull from C with a left ventrolateral trachea.

The ring-billed gull (*Larus delawarensis*) also displayed a greatly dorsolaterally placed trachea and esophagus. Only in the very most cranial region of the neck (first 1 cm) were the two only laterally, and not dorsolaterally, situated (Fig 9). They enter the thoracic inlet while still considerably dorsally placed. In one of the three dissected individuals (Fig 9C and 9D), the trachea was ventrolaterally situated on the left side and had practically no rotational component. The esophagus passed along the neck’s right ventrolateral side in this specimen.

#### Order Accipitriformes; Order Strigiformes; Order Coraciiformes; Order Piciformes; Order Falconiformes

**Fig 10:**
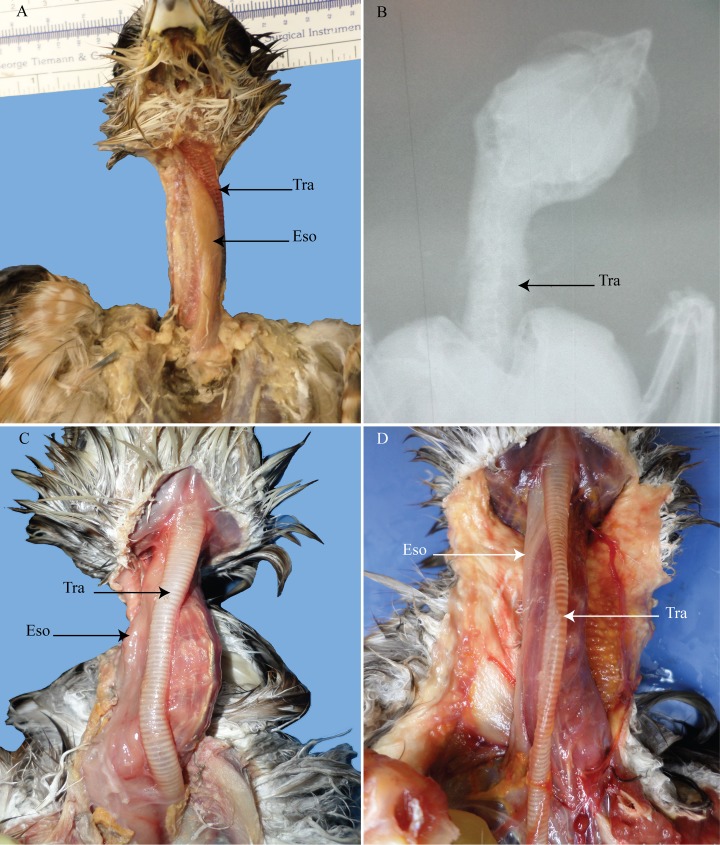
Accipitriformes. (A) *Accipiter cooperii* placement of the trachea and esophagus are on the left side of the neck. (B) radiograph of *A*. *cooperii* also showing the trachea on the left. (C) *Buteo jamaicensis* ventral view showing a more typical right lateralization. (D) *Buteo platypterus* ventral view displaying a midsagittal position of the trachea. The trachea is also twisted nearly 180°. Tra = trachea; Eso = esophagus.

Cooper’s hawk (*Accipiter cooperii*) was very similar to most birds in having both the trachea and the esophagus displaced together. However, unlike the majority of birds observed, which have the trachea and the esophagus lateralized on the right side of the neck, in the Cooper’s hawk these organs were displaced together on the left lateral side of the neck making an almost immediate lateral placement from exiting the oropharynx. The trachea lay farther left laterally and surprisingly dorsally to the esophagus, which was situated nearer to the vertebrae (Fig 10A and 10B). Near the entrance to the thoracic cavity, the esophagus became situated more along the midline of the body. The trachea, having been rotated to lie on its side beyond the lower cranial section, entered the thorax too while on its side. One individual of the red-tailed hawk (*Buteo jamaicensis*), like Cooper’s hawk, had a trachea situated left laterally that had rotated to a large extent. The short esophagus remained more along the midline until it became a largely inflated crop which cut transversely left to cover (be situated ventral to) the trachea. The trachea could be on the left in this individual due to the large crop having pushed it over. In a second individual (Fig 10C), the trachea and esophagus were situated right lateral, and tracheal rotation was only minor. Perhaps the most interesting configuration observed in hawks came from the broad-winged hawk (*Buteo platypterus*). The broad-winged hawk had a right laterally placed esophagus with a midsagittal placement of the trachea. Far more importantly, though, the trachea was actually twisted so that the dorsal side faced ventrally and vice versa (Fig 10D). The twisting of this trachea occurs very close to its origin from the oropharynx. It stays twisted for most of its length. It is only at the entrance to the thoracic cavity that it untwists. This bizarre twisting is speculated here to be the result of the trachea (mobile in birds as it is) having been moved from its original left lateral position to a midsagittal position (and perhaps beginning to become right lateral). This might explain why the twist makes the dorsal side of the trachea face ventrally.

As tracheal rotation makes the mediodorsal point of the trachea face inward toward the vertebral complex, the dorsal side (now facing ventrally) with a counterclockwise rotation would indicate an initial left lateral placement. This is because as the dorsomedial point should face toward the vertebrae, a counterclockwise (left rotated) rotation points the mediodorsal point right (and so if the trachea were situated right lateral with counterclockwise rotation, the mediodorsal point would face right, or outward rather than inward). Therefore, it is likely that the broad-winged hawk had an initial tracheal placement on the left side of the neck. Ultimately, this peculiar incidence is likely nothing more than an aberration.

The great horned owl (*Bubo virginianus*), the barred owl (*Strix varia*), and the Eastern screech owl (*Megascops asio*) all exhibited right lateral displacement of both the esophagus and trachea with little to no tracheal rotation. The same was observed in the belted kingfisher (*Megaceryle alcyon*), the yellow-bellied sapsucker (*Sphyrapicus varius*), and the American kestrel (*Falco sparverius*).

#### Order Psittaciformes; Order Passeriformes

The only extant psittacid examined in this study came from a CT scanned Hispaniolan Amazon (*Amazona ventralis*). The trachea could clearly be seen on the right side of the neck. Unfortunately, no image of the esophagus is seen. However, King and McLelland ([29], p. 91) do illustrate the internal anatomy of a budgerigar (*Melopsittacus undulatus*) detailing that the esophagus lies on the right side as well. In addition, the trachea is illustrated to lie between the esophagus and the muscles of the neck while also lying behind the transversely oriented crop. It essentially follows the pattern observed in the prairie chicken.

Various Passeriformes were dissected and observed including: the barn swallow (*Hirundo rustica*), the brown creeper (*Certhia americana*), the American robin (*Turdus migratorius*), the hermit thrush (*Catharus guttatus*), the cedar waxwing (*Bombycilla cedrorum*), the ovenbird (*Seiurus aurocapilla*), the common yellowthroat (*Geothlypis trichas*), the Canada warbler (*Wilsonia canadensis*), the dark-eyed junco (*Junco hyemalis*), the northern cardinal (*Cardinalis cardinalis*), the rose-breasted grosbeak (*Pheucticus ludovicianus*), the red-winged blackbird (*Agelaius phoeniceus*), the common grackle (*Quiscalus quiscula*), the western meadowlark (*Sturnella neglecta*), the house finch (*Carpodacus mexicanus*), and the American goldfinch (*Spinus tristis*). In all of these birds, the trachea and esophagus were joined together and situated on the right side of the neck. Together, the two diverged onto the right side nearly immediately after the exiting the oropharynx. The trachea was rotated onto its side in these birds most commonly only to a minimal extent.

### Mammals

#### Class Mammalia; Order Pilosa

**Fig 11:**
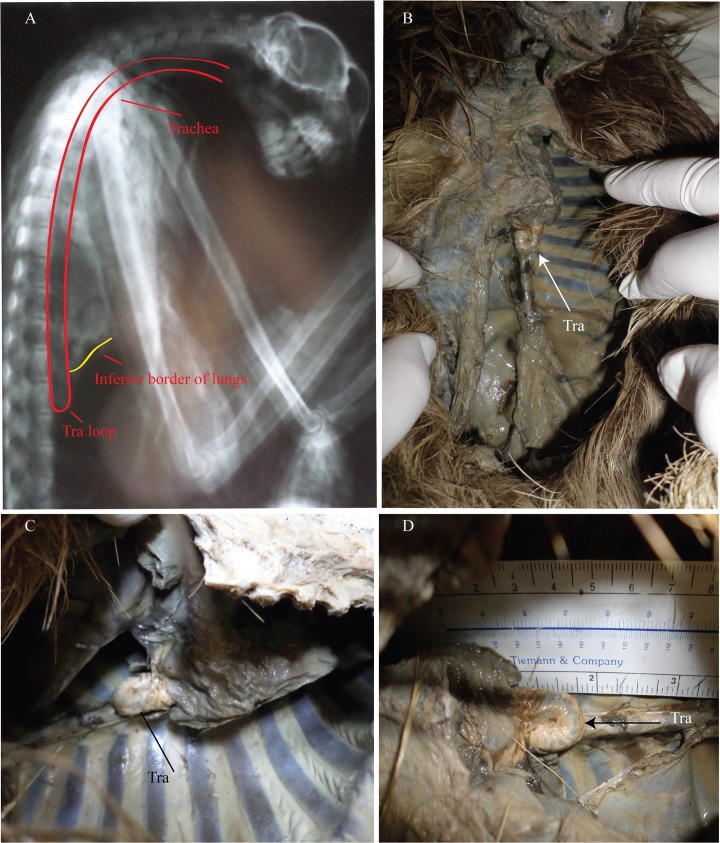
*Bradypus tridactylus* views of the tracheal loop. (A) lateral radiograph of the course of the trachea. (B-D) views of the tracheal loop. Tra = trachea; Tra loop = tracheal loop.

The trachea of the three-toed sloth (*Bradypus tridactylus*) is exceptionally long. There are 100 tracheal rings [30–39]. It is long enough to reach the diaphragm. Upon reaching the diaphragm, it curves, forming a U (Fig 11), then proceeds anteriorly for 2–4 cm, and then yet again curves ventrally for about a centimeter before opening into a sac [40]. The loop is 36 mm long [34]. The tracheal elongation exhibited by *Bradypus* is unique among mammals [33]. Within the chest, the trachea is dorsally situated as it courses along and behind the lungs. Tracheal elongation even differs ontogenetically within the species. In embryos, the tracheal loop is placed proximally to the lungs [31]. The trachea then proceeds dorsally along the lungs where it passes to the distal end of the lungs before branching into bronchi which branch cranially into the lungs [31]. The tracheal width at the loop was about 5.7 mm with its dorsoventral diameter at 5.5 mm. In the neck, the tracheal width was about 8.1 mm. Unlike *Bradypus*, the two-toed sloth, *Choloepus hoffmanni*, has a shorter neck (at six cervical vertebrae compared with *Bradypus*’ eight–nine), with a much more limited range of mobility and a shorter trachea [40]. Personal observations of *Choloepus hoffmanni* confirmed a normally situated and non-looped trachea.

It would seem that, in general, the mammalian condition is the standard normalized condition. This has been observed in further personal dissections of mice (*Mus musculus*), cats (*Felis catus*), dogs (*Canis familiaris*), pigs (*Sus scrofa*), and horses (*Equus ferus caballus*). Even among other sloths and other xenarthrans, the three-toed sloth is unique in having elongation and looping [40].

Table 2 below summarizes all personally dissected specimens (and some data of personal observations and reports from the literature), their conditions, and other relevant and pertinent observations related to tracheal and esophageal visceral geography.

**Table 2:** Summary of Vertebrate Tracheal and Esophageal Positions from Dissections and Personal Observations.

Taxa	Condition	Placement of Organs	Immediacy of Displacement	Degree of Tracheal Rotation
**Chelonia**				
*Pseudemys concinna*	normal	midsagittal	-	-
**Lacertilia**				
*Anolis carolinensis*	normal	midsagittal	-	-
**Crocodylia**				
*Alligator mississippiensis*	normal	midsagittal	-	-
*Crocodylus niloticus*	displaced	esophagus in midline; trachea on left side	quickly	(not known)
**Aves**				
*Struthio camelus*	displaced	both right lateral	gradual	minimal
*Branta canadensis*	displaced	both right lateral	gradual	minimal
*Branta sandivicensis*	displaced	both right lateral	gradual	(not known)
*Aix sponsa*	displaced	both right lateral	rather quickly	large
*Anas platyrhynchos*	displaced	both right lateral	rather quickly	large
*Tympanuchus cupido*	displaced	both right lateral	gradual	minimal
*Meleagris gallopavo*	displaced	both right lateral	gradual	none
*Columba livia*	displaced	separated (eso on right, tra on left)	gradual—immediate	minimal—large
*Zenaida macroura*	displaced	separated (eso on right, tra on left)	gradual—immediate	large
*Fulica americana*	displaced	both right lateral	gradual	none—large
*Rallus limicola*	displaced	both right lateral	immediate	moderate
*Coccyzus americanus*	displaced	both right lateral	rather quickly	none
*Phalacrocorax auritus*	displaced	•both right lateral •eso right lateral, tra midline	rather quickly	moderate
*Ardea herodias*	displaced	both right lateral	rather quickly	large
*Larus delawarensis*	displaced	•separated (eso on right, tra on left) •both right lateral	immediate	none—large
*Buteo jamaicensis*	displaced	•separated (eso on right, tra on left) •both right lateral	immediate	none—large
*Buteo platypterus*	displaced	esophagus right lateral; trachea midline	immediate	large
*Accipiter cooperii*	displaced	both left lateral	immediate	large
*Bubo virginianus*	displaced	both right lateral	immediate	none
*Strix varia*	displaced	both right lateral	immediate	minimal
*Megascops asio*	displaced	both right lateral	immediate	minimal
*Megaceryle alcyon*	displaced	both right lateral	immediate	minimal
*Sphyrapicus varius*	displaced	both right lateral	immediate	moderate
*Falco sparverius*	displaced	both right lateral	immediate	none
*Amazona ventralis*	displaced	both right lateral	immediate	(not known)
*Melopsittacus undulatus*	displaced	both right lateral	immediate	(not known)
*Hirundo rustica*	displaced	both right lateral	immediate	minimal
*Certhia americana*	displaced	both right lateral	immediate	none
*Turdus migratorius*	displaced	both right lateral	immediate	large
*Catharus guttatus*	displaced	both right lateral	immediate	none
*Bombycilla cedrorum*	displaced	both right lateral	immediate	large
*Seiurus aurocapilla*	displaced	both right lateral	immediate	minimal—large
*Wilsonia canadensis*	displaced	both right lateral	immediate	minimal
*Geothlypis trichas*	displaced	both right lateral	immediate	minimal
*Junco hyemalis*	displaced	both right lateral	immediate	minimal
*Pheucticus ludovicianus*	displaced	both right lateral	immediate	minimal
*Cardinalis cardinalis*	displaced	both right lateral	immediate	minimal
*Sturnella neglecta*	displaced	both right lateral	rather quickly	minimal
*Quiscalus quiscula*	displaced	both right lateral	immediate	minimal
*Agelaius phoenicius*	displaced	both right lateral	immediate	minimal
*Carpodacus mexicanus*	displaced	both right lateral	immediate	moderate
*Spinus tristis*	displaced	both right lateral	immediate	minimal
**Mammalia**				
*Bradypus tridactylus*	normal	midsagittal	-	-

### Anatomical structuring allowing for displacement

The most important factor allowing for displacement of the trachea and/or esophagus is the cervical muscular anatomy. The arrangement of jaw and neck muscles dictates how an animal feeds [41]. It also dictates the relative degree of mobility of these organs.

The trachea and esophagus of the turtle are well constricted and held firmly in place by both connective tissue and muscles. Muscles which constrict these organs on the ventralmost periphery include the *m*. *mylohyoideus* at the laryngeal level, and the *m*. *latissimus colli* for the rest of the length of the neck. Deep to the *m*. *latissimus colli* lie three additional muscles, *m*. *sternohyoideus*, *m*. *omohyoideus*, and *m*. *sternomastoideus*. These constrict the trachea and esophagus both ventrally and laterally. Nomenclature of the turtle neck follows Ashley [42]. The *m*. *latissimus colli* originates on the sides of the cervical vertebrae, inserting with the other half on the midventral line, and acts to compress the throat in swallowing [42]. The *m*. *sternomastoideus* inserts on the base of the skull, having originated on the ligament at the tip of the posterior hyoid [42]. The connective tissue that holds the esophagus to the vertebral muscles holds it very firmly to it, unlike that of birds. However, despite these muscular constraints some turtles have been observed with displaced tracheae.

In lizards, restriction is due to the muscles *m*. *omohyoideus*, the m. episternohyoid complex consisting of *m*. *sternohyoideus* and *m*. *constrictor colli*, and the *m*. *episternocleidomastoideus*. Terminology follows Jones et al. [41] for their assessment of muscles of basal diapsids and *Sphenodon*. The *m*. *constrictor colli* passes along the anterior and mid-neck regions, attaching to the muscle fascia of the posterior portions of the *m*. *depressor mandibulae* [43–49]. The *m*. *omohyoideus* originates from the scapulocoracoid ligament in addition to the anteromedial portion of the scapula [50]. It inserts on the ventral surface of the posterior portion of the basihyal and the posteroventral edge of the proximal portion of the first ceratobranchial [51]. The *m*. *sternohyoideus*, which lies deep to the previous muscle, originates on the episternum and the anterior surface of the medial region of the clavicle [50]. It inserts on the posterodorsal surface of the distal part of the first ceratobranchial [51, 52]. The episternocleidomastoid originates from the anterior margin of the clavicle and interclavicle [48–50, 53]. Recently, Al-Hassawi [48, 49] described three branches of insertions: branch one inserts on the posterolateral end of the paroccipital process of the opisthotic, branch two inserts on the posteromedial margin of the squamosal above branch one, and branch three inserts on the posterodorsal edge of the squamosal and parietal.

Given the ubiquity of tracheal and esophageal displacement in Archosauria, in spite of the vast overall morphological differences between Crocodylia and Aves, it is critical to understand the differences between the necks of these groups. The Canada goose (*B*. *canadensis*) served as a model organism to detail muscularization in birds because it is common and of large size, which facilitated distinguishing of muscles. The American alligator (*A*. *mississippiensis*) served as a model organism for Crocodylia. It is suspected that aves are so commonly seen to display tracheal and esophageal displacement because of the many modifications of the muscles in their cervical regions. In crocodylians, scapular and infrahyoid muscles were primarily responsible for restricting or limiting the degree of mobility of the trachea and/or esophagus.

**Fig 12:**
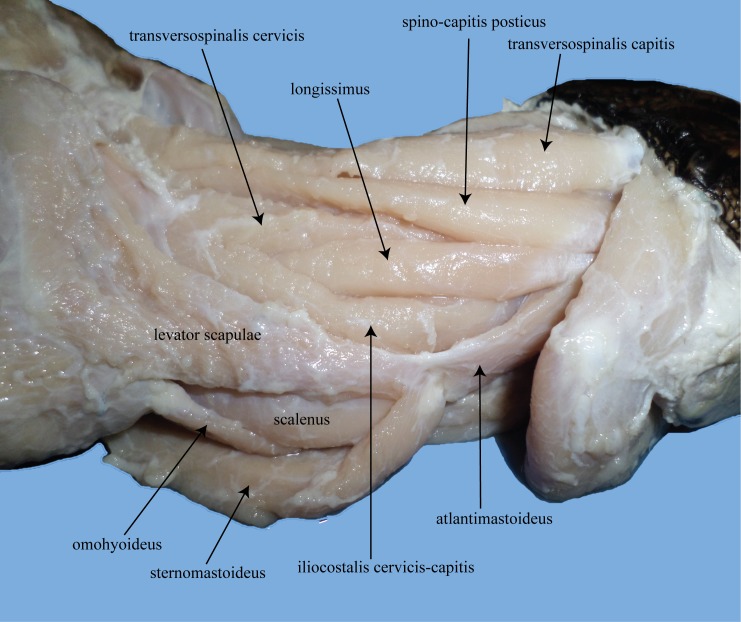
Lateral view of the cervical musculature of *Alligator mississippiensis*. *M*. *sternomastoideus* is reflected away ventrally to expose the *m*. *omohyoideus* and *m*. *scalenus*.

**Fig 13:**
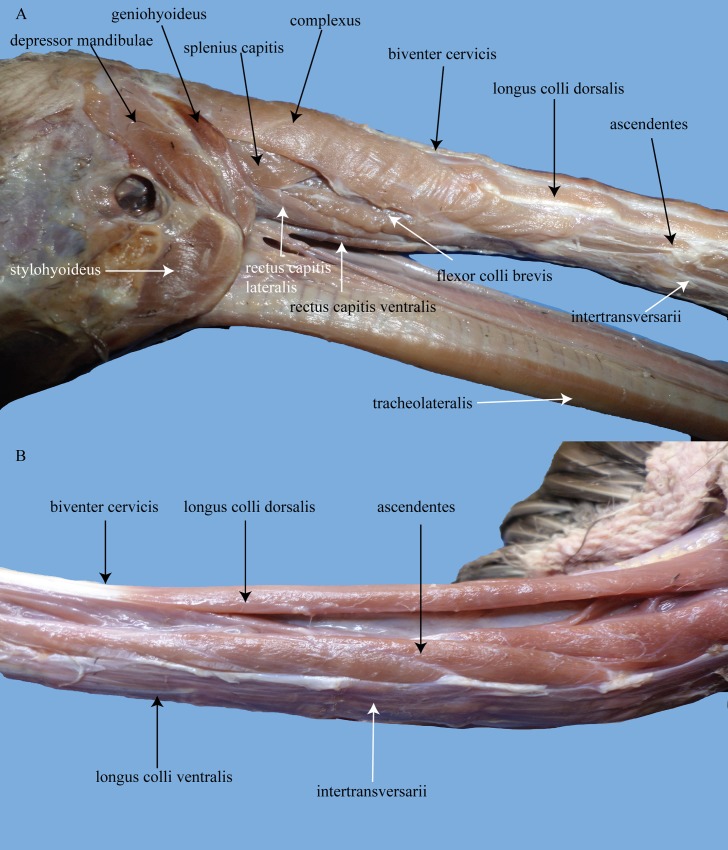
Lateral views of the cervical musculature of *Branta canadensis*. (A) anterior muscles. (B) posterior muscles.

**Fig 14:**
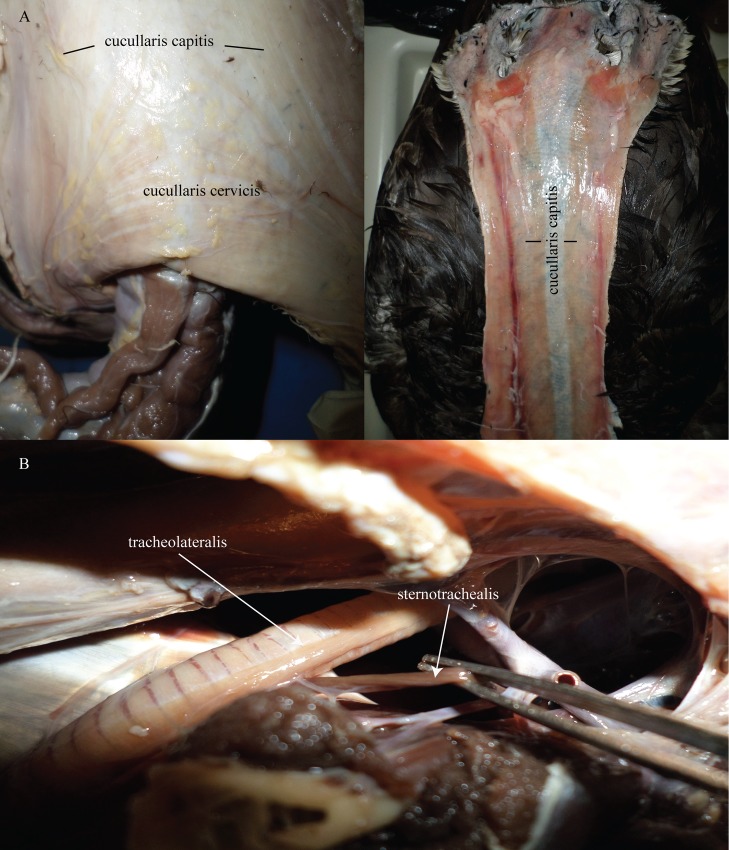
Cucullaris and tracheal muscles of *Branta canadensis*. (A) Left: dorsal view of the posteriormost portion of the neck with the skin peeled back to reveal the *m*. *cucullaris cervicis* and *capitis*. Right: dorsal view of the skin of the neck peeled back to display the *m*. *cucullaris capitis*. (B) view of the inside of the thorax to show the m. tracheolateralis and m. sternotrachealis of the goose.

In birds, in contrast, scapular and infrahyoid muscles have become lost, reduced, or modified. For instance, the crocodylian *m*. *sternomastoideus* is part of a larger muscle complex, the *m*. *cucullaris*. The *m*. *cucullaris* of crocodylians can be divided into the *m*. *dorsoscapularis* and *m*. *capitisternalis* [53]. The *m*. *dorsoscapularis* originates on the anterior edge of the proximal part of the scapula and inserts on the dorsal fascia in the midline of the posterior cervicals [54, 55]. The *m*. *capitisternalis* is itself further divided into an anterior portion, the *m*. *atlantimastoideus*, and a posterior portion consisting of the *m*. *sternoatlanticus* (here *m*. *sternomastoideus*) + *m*. *levator scapulae* (Fig 12); [53]. The *m*. *atlantimastoideus* originates from the rib of C1 or C2, depending on species, and inserts on the distal and ventral margins of the paroccipital process [53]. The *m*. *sternoatlanticus* originates from the anterior margin of the ventral surface of the sternum and inserts on the tip of the rib of C1 or C2 [53]. In aves, *m*. *cucullaris* also consists of two portions (Figs 13 and 14). The first portion is the *m*. *cucullaris capitis*, or the *m*. *dermotemporalis* of George and Berger [56], which lies on the lateral to ventrolateral surface of the neck [53]. The origin of the slips of this muscle include clavicle, ligament which stretches between the clavicle, coracoid, and sternum (Baumel and Raikow’s [57] *membrana sternocoracoclavicularis*), and/or skin [58, 59]. The muscle inserts on the occipital region of the skull [53]. The second portion, the *m*. *cucullaris cervicis*, lies dorsal to the aforementioned muscle, arising from the clavicle and inserting on the mid-dorsal raphe of the most posterior cervical vertebrae [58]. Fürbringer [60] homologized the avian *m*. *cucullaris capitis* to the crocodylian *m*. *capitisternalis* and the avian *m*. *cucullaris cervicis* to the crocodylian *m*. *dorsoscapularis*.

The *m*. *cucullaris*, as just described above, is what creates, partially, the restrictive muscles in the crocodylia and likewise the homologous musculature in aves. The difference, however, is that in birds, these restrictive muscles have become dermally attached muscles rather than actually attaching to the axial and/or appendicular skeleton directly (Fig 14 above). The cucullaris is not a true muscle of the axial skeleton [56]. In so doing, the once thick, restrictive muscles have now become modified into thin, dermally attached coverings which exert less control/hold on the location of the organs.

**Fig 15:**
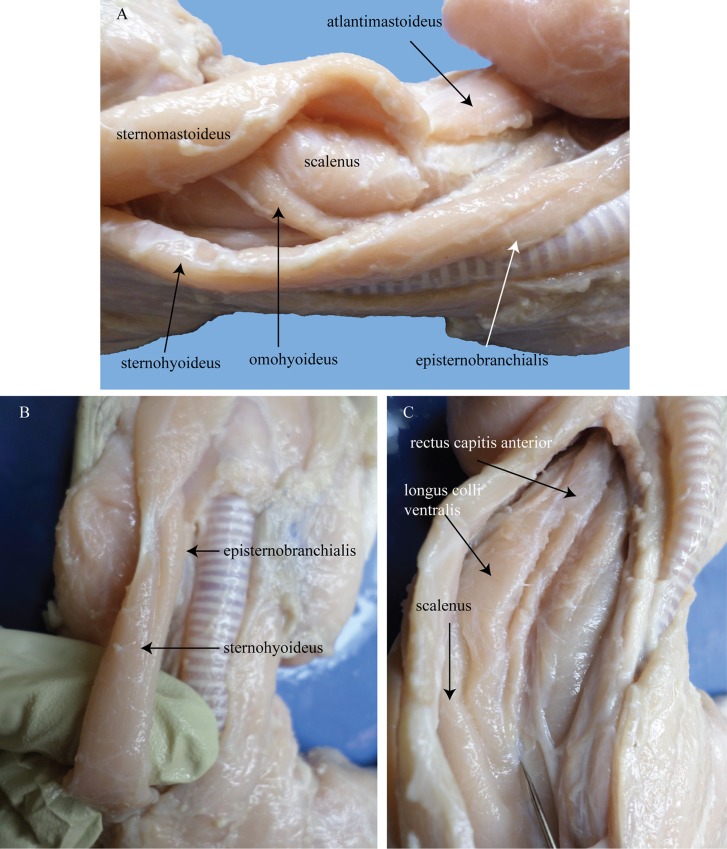
*A*. *mississippiensis* cervical and infrahyoid musculature. (A) lateral view of the neck and infrahyoid musculature. (B) ventral view of the *m*. *episternobranchialis* and *m*. *sternohyoideus*. (C) ventral view of the ventralmost cervical muscles with the trachea, esophagus and infrahyoid muscles pushed aside.

Aside from the *m*. *sternomastoideus* (*m*. *sternoatlanticus*), the alligator esophagus and trachea are bordered by several other muscles. The esophagus is bordered dorsally by the *m*. *rectus capitis anterior*, *m*. *longus colli ventralis*, and the *m*. *scalenus* (Fig 15C). Ventrally, the trachea is covered by the *m*. *episternobranchialis* (Fig 15A and 15B) which originates from the craniolateral edge of the anteromedially projecting episternum and inserts on the front, posterior part of the horn of the hyoid. It is a narrow and flat muscle which lies also a bit laterally to the trachea as well. In *A*. *mississippiensis*, the *m*. *sternohyoideus* (Fig 15A and 15B) was observed to originate similarly from the craniolateral process of the episternum, immediately caudolateral to the *m*. *episternobranchialis*. The *m*. *sternohyoideus* is a flat, broad muscle coursing ventrally down the length of the neck covering the ventrolateral portion of the trachea. Near the posterior portion of the hyoid it forms a short, strong tendon, while anteriorly it inserts onto the posterodorsal margin of the splenial. The *m*. *omohyoideus/m*. *coracohyoideus* was observed to be a long, narrow, string-like muscle originating at the upper border of the coracoid near the scapula traveling along the ventral/ventrolateral region of the neck coursing beside the length of the esophagus and dorsal to the *m*. *sternohyoideus* and inserting onto the middle of the dorsal side of the horn of the hyoid.

Unlike crocodylians, birds also have tracheal and syringeal muscles. The observed extrinsic tracheal muscles of the Canada goose included *m*. *sternotrachealis*, *m*. *cleidotrachealis*, and *m*. *tracheolateralis* (Fig 14B). These muscles are responsible for tracheal movement [27]. The *m*. *cleidotrachealis* may be a specialized portion of the sternohyoid-sternotrachealis complex [56]. Gadow and Selenka [61] considered *m*. *tracheohyoideus*, *m*. *thyrohyoideus*, *m*. *sternotrachealis* and *m*. *cleidotrachealis* to be derived from a primitive sternohyoid layer, which may still be present in a few birds (*Apteryx* and parrots).

The alligator *m*. *constrictor colli* is divided into anterior and posterior portions, with the former arising by a tendinous aponeurosis from both the first superficial tendon aponeurosis of the *m*. *pterygoideus posterior* and posteriorly from the dorsal aponeurosis [62]. It passes ventrally around the neck, attaching to a thin tendon plate or partly into the ventral cervical fascia [62]. The *m*. *constrictor colli profundus* of alligators arises laterally from the first two cervicals, continues ventrally around the pharynx and very uppermost trachea, and inserts on the ventral surface of the hyoid apparatus [62]. It is a pharyngeal constrictor. A superficial layer immediately deep to the skin of the neck of the avian *m*. *cucullaris* is composed of circularly arranged fibers which function as the constrictor colli [56]. Finally, of course there are many more tongue and hyoid muscles in alligators and birds. However, they are not discussed here as they only hold the hyoid apparatus in place and have little or no influence on the position of the trachea and esophagus along the length of the neck.

### Comparisons of tracheal and neck lengths

Measurements of the length of the neck and trachea of several species of birds revealed significant differences in tracheal versus neck lengths (Table 3). The difference between the length of the neck and the length of the trachea (in neutral posture) differed substantially from the stretched posture measurements in most cases. On average, the percent difference in neck and tracheal measurements for the neutral posture was 25% whereas the stretched difference was 5%.

**Table 3:** Differences in Neck and Tracheal Lengths in Birds.

Species	Length of Neck (mm)	Length of Trachea (mm)	Percent L(tra) to L(neck)	Percent Differences in Length
*Aix sponsa*	119.4	82	68%	32%
Aix sponsa*	123.1	120.7	98%	2%
*Anas platyrhynchos*	155.6	130.7	84%	16%
Anas platyrhynchos*	156.7	150	96%	4%
*Phalacrocorax auritus*	183.2	128.3	70%	30%
Phalacrocorax auritus*	205.9	201.2	97%	3%
*P*. *auritus baby*	75	58.7	78%	22%
*P*. auritus baby*	76.3	75.9	99%	1%
*Rallus limicola*	65.2	34.1	52%	48%
Rallus limicola*	66	59.9	90%	10%
*Falco sparverius*	62.3	48.6	78%	22%
Falco sparverius*	62.6	57.6	92%	8%
*Strix varia*	81.4	80.6	99%	1%
Strix varia*	82.3	81.5	99%	1%
*Seiurus aurocapilla*	40.8	29.5	72%	28%
Seiurus aurocapilla*	60.2	57.1	94%	6%
*Turdus migratorius*	43.7	39.7	90%	10%
Turdus migratorius*	44.1	43.6	98%	2%
*Cardinalis cardinalis*	41.7	31.4	75%	25%
Cardinalis cardinalis*	42.1	36.6	87%	13%
*Bombycilla cedrorum*	36.3	22.5	61%	39%
Bombycilla cedrorum*	37.1	33.5	90%	10%

(*) Denotes a stretched posture measurement.

## Discussion

### General trends and observations of tracheal and esophageal displacement

#### Positions, degree and immediacy of displacement

Birds demonstrate tracheal and esophageal displacement with notably lateralized tracheae and esophagi. Unlike the trachea and esophagus which may shift their positions, the rest of the cervical viscera do not move to one side or other. Rather, the arteries, veins and nerves occupy their respective sides (i.e., the left external carotid occupies the left side of the neck whereas the right external carotid stays on the right side). Of the 42 species of extant birds dissected/observed/reported in this study, the most ubiquitous pattern seen was right lateralization of the trachea and esophagus together. Two other less common patterns observed were left lateralization together and separated lateralization (where the trachea lay on one side whereas the esophagus lay on the other side). Additionally, displacement was most commonly seen to be achieved immediately upon leaving the oropharyngeal region. There, they would then remain lateral for the entire length of the neck, only rejoining the midline at the most immediate region of the thoracic cavity. Immediate lateralization is especially apparent in smaller and shorter necked birds such as the passerines and owls. However, sometimes the lateralization was slight and/or reached gradually. As this suggests, this is when one or both of the organs actually is on the midline and only gradually becomes asymmetrical. In these instances, the organs become displaced notably, or only, in the posterior cervical region. The gradual type of displacement occurred predominantly in the larger bodied, longer necked birds (e.g., *Branta canadensis*, *Fulica americana*, *Meleagris gallopavo*, and *Struthio camelus*). It is certainly not impossible for the trachea to lie on the ventral midline of the neck, and this was observed sometimes (e.g., juvenile *Phalacrocorax auritus* and *Buteo platypterus*). Unlike Snively’s [10] comment which stated that only in the most caudal region of the neck does the trachea divert from the midline, it has been shown here that the trachea and esophagus may divert to an asymmetrical position at any region of the neck.

**Fig 16:**
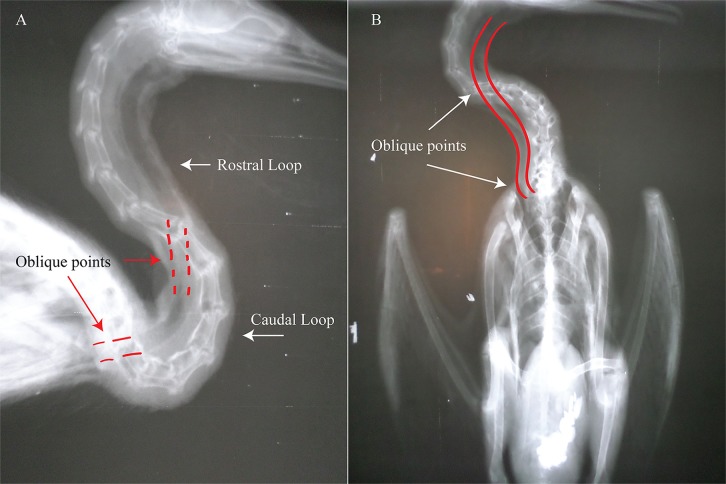
Radiographs of *P*. *auritus* neck. (A) “Rostral loop” is the upper half of the “S” shape of the bird neck; while the “caudal loop” is the lower half. Terminology after Van der Leeuw et al. [63]. Lateral view showing the course of the trachea and its oblique points (point where the trachea crosses planes (medial to lateral and vice versa). (B) ventral view showing the course of the trachea and its oblique points.

Though tracheae are predominantly observed to be preferentially on the right side of the neck, the trachea remains a highly mobile organ capable of a dynamic array of varied positions both inter and intraspecifically. This was particularly evident with *Larus delawarensis* and *Buteo jamaicensis*. In *Larus*, while the trachea in two specimens was observed to be right lateral, the trachea in one was ventrolateral along the left side of the vertebral column. In *B*. *jamaicensis*, one individual exhibited right lateral placement of both organs. In another, a right lateral esophagus with a left lateral trachea occurred. In this individual, the trachea may have been situated left lateral due to a hugely inflated crop which probably pushed the trachea to the other side. Further, positioning may be variable to the extent not only of which side the organs occupy but how immediately asymmetry is approached. Take for instance the rock pigeon, *C*. *livia*. Within *Columba*, these organs were displaced in some individuals immediately whereas others had a trachea which did so gradually (see Fig 5). The presence of a crop might also be a major factor influencing position. The large, bilobed crop of the pigeon, which cuts transversely across the lower neck, seems to cause the trachea to be pushed dorsolaterally onto the side of the neck counterlateral to the esophagus. The same can be said of the mourning dove and the red-tailed hawk. However, this does not explain why the trachea remains as close as it does to the esophagus and crop of the prairie chicken and parrots. Due to a great range of mobility, the trachea may be placed ventrolaterally or mid-laterally. Additionally, the trachea and esophagus may be dorsolateral. Dorsolateral placement of the organs is, essentially, nothing more than the result of the organs cutting past a highly S-shaped neck (Fig 16A). In particular, it is because the organs cut across the “caudal loop” (terminology after Van der Leeuw et al. [63]) of the S of the cervical column. This crossing dorsal to the caudal loop is obvious in the great blue heron, *Ardea herodias*, where the organs are situated dorsally for most of their length as the “rostral loop” is considerably shortened in comparison to the caudal loop. What plane (ventral, mid, or dorsal) the organs occur in may vary regionally, can shift suddenly, and can fluctuate along the neck. The S-shape of the avian neck is due to a comparatively long neck with 12–24 cervical vertebrae with highly dorsoventrally flexible saddle-shaped joints [63]. The trachea and esophagus, more or less, make the straightest possible path from their origins to their termini, avoiding the problem of having to follow an S curve (which due to the two loops/curves has greater surface area than a straightened neck). Tracheae are held in a firm but less than completely taut manner. This, plus the elastic annular ligaments which join the tracheal rings to one another, allows for the trachea to have some slack.

#### Oblique

As displacement occurs, the trachea and esophagus move obliquely (oblique is defined here as having crossed two planes, medial/sagittal to lateral/parasagittal and vice versa). The transition from these planes occurs commonly both near the oropharynx and near the base of the neck/entrance of the thorax (Fig 16A and 16B). It is typically slight, as the width of the bird neck is thin. As the neck is so thin, the chances of and ability of the trachea and esophagus to achieve displacement are further increased. Depending on species, there may occasionally also be a dorsoventral component to this oblique movement. That is, aside from moving from a lateral to medial plane, the trachea may also just slightly cross from a dorsal to a slightly less dorsal plane. The dorsoventral component is the result of a dorsolaterally situated trachea (which was itself due to cutting through the caudal loop) cutting across and down.

#### Rotation

**Fig 17:**
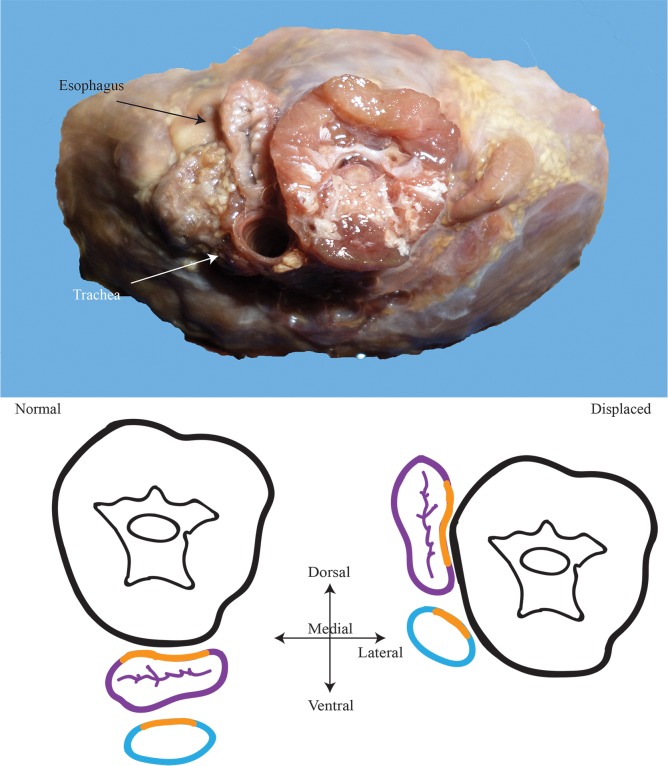
Rotation of the organs. Top: bird neck cut to show the extent of rotation of the trachea and esophagus. Bottom: Illustrations of normality and displacement. Bottom left: an illustration of the normal, midsagittal, condition. Bottom right: an illustration of tracheal and esophageal displacement. Rotation is evident. Orange lines outline the mid-dorsal portions of the organs. In TED, the organs can be seen to have been rotated so that their mediodorsal points face inward toward the vertebral complex.

Another important and predominant observation was tracheal rotation. The trachea frequently rotates onto its side so that its mediodorsal point is almost always facing inward, toward the vertebral muscles (Fig 17). In so doing, the lateral sides of the trachea become dorsoventral while the dorsal and ventral sides of the trachea face mediolaterally. This is particularly obvious for those birds which have ovoid tracheae. Even for those with circular tracheae, where it might otherwise be difficult to distinguish sides, rotation can still be obviously seen by looking at the *m*. *tracheolateralis* muscles which border the left and right sides of the trachea. Additionally, in keeping with the typical vertebrate arrangement, the esophagus still remains dorsal to the trachea and actually appears, itself, to have been rotated onto its side as well. Most avian tracheae are rotated; however, it is not always the case. In a number of short-necked birds (e.g., *Catharus guttatus*, *Bubo virginianus*, *Strix varia*, and *Coccyzus americanus*) the trachea either completely lacked rotation or exhibited only slight rotation. Rotation might be due to the fact that the trachea and esophagus, while only loosely anchored, are still attached to the vertebral muscles. This attachment may provide a minor constraint so that while they can move off to the side, the connection keeps them from otherwise jutting out horizontally. Upon entering the thoracic cavity, the trachea will also rotate back to a normal orientation.

#### Normality

Normal, midsagittal pathways differ in a number of respects from the laterally displaced condition. The normal path follows the typical vertebrate condition where the esophagus lies ventral to the vertebral column but dorsal to the trachea. In lateral profile, both the esophagus and the trachea pass ventrally, paralleling the vertebral column along its path. In ventral profile, both organs will, of course, lie in the ventral midline with essentially only the trachea visible (Fig 17). Additionally, when viewed parasagittally, only one side of the trachea (right or left) will be visible. Unlike the lateral condition in which the trachea and esophagus may enter the thoracic cavity ventromedially, ventrolaterally, midlaterally, or, to a degree, dorsolaterally, the normal condition involves the two entering the thoracic inlet only midsagittally. Animals which possess a normalized route do not exhibit tracheal rotation, nor do their organs cross between medial and lateral planes.

### Function of displacement

The lengths of the trachea versus neck, seen in Table 3, indicate significant differences in the lengths of the neck and trachea of birds when stretched and neutral. Due to the largely S-shaped curve of the avian neck, and the fact that tracheae most often head along a straight trajectory across the side of the neck (thus, creating the shortest possible pathway), this is unsurprising. As the neck is curvilinear, the curves of the neck (i.e., the “rostral” and “caudal” loops) create added surface area and length. Because the avian neck has few muscular constraints, the cervical viscera may slide feely to one side. In doing this, the trachea becomes simultaneously straight and relaxed (unstretched) as it avoids the bends and extra length. Thus, when the neck is extended forward, there is more available tracheal length to stretch to than if it were forced to conform to curves which, necessarily, would keep the trachea in a more unrelaxed/stretched state. Therefore, lateralization is useful in allowing slack, or added tracheal (and esophageal) extension, to keep up with the demands of the highly flexible and extendable avian neck. This is further evident when comparing the relaxed neck and tracheal lengths to the stretched neck and tracheal lengths. When stretched, the trachea extends to reach a length almost exactly equal to that of the neck. This is possible because the annular ligaments joining the tracheal rings together allow for some accordion-like properties to stretch and compress the trachea. Further, this creation of the shortest possible path to the lungs may also reduce tracheal dead space. Perhaps an additional explanation for displacement is due to feeding. As birds do not masticate, but rather swallow prey items whole, this creates a larger sized bolus. A loosely attached, mobile esophagus and trachea capable of moving out of one another’s way would certainly be useful to accommodate each other’s space and allow for the passage of a large bolus that would simply push the trachea away without crushing the tracheal rings.

The issue of why mammals do not achieve tracheal and esophageal displacement is two-fold. First, muscles such as *m*. *sternohyoideus*, *m*. *sternothyroideus*, *m*. *sternocleidomastoideus*, and *m*. *omohyoideus* inadvertently confine the trachea and esophagus to a midsagittal position and restrict large movements. Second, the short length of the mammalian neck simply does not leave much length for these organs to deviate from the midline. Short necks and infrahyoid and scapular/strap muscles do act as relatively powerful constraints; however, they do not always constrain position, as was seen in crocodilids and some turtles. Straight tracheae and esophagi can, occasionally, overcome these morphological restrictions and lateralize yet it remains unknown among mammals.

One final interesting point is that 60 species of birds have been observed to have tracheal elongation [28]. An elongated trachea was also observed in *B*. *canadensis* and *B*. *sandvicensis*. Therefore, 62 species of birds exhibit it, and it is very much possible that many other species of birds, not yet thoroughly examined, also possess an elongated and looped trachea.

## Conclusions

The trachea and the esophagus can be commonly seen to be asymmetrically placed along the length of the neck in crocodilids, perhaps gavialids, and also overwhelmingly in aves. While alligators are not characterized by this trait, it is possible for the trachea to be displaced in the posterior region, if not only circumstantially. Turtles and snakes, like crocodilids, may variably demonstrate the character and overcome seemingly restrictive muscles to do so. Mammals and lizards do not seem to display this trait. The most commonly observed form of tracheal and esophageal displacement involves the trachea and esophagus coursing right lateral along the length of the neck with a fair degree of tracheal rotation. The trachea may be ventrolateral, midlateral, and dorsolaterally positioned depending on the region of the neck in which the trachea occurs. Degree of laterality and position may change abruptly.

The most important physical constraint to tracheal and esophageal displacement is musculature. Of the muscles spanning the neck, the most important are those that constrict tracheal and esophageal movements ventrally and laterally such as *m*. *sternomastoideus* (episternocleidomastoid), *m*. *sternohyoideus*, *m*. *episternobranchialis*, and *m*. *omohyoideus*. The highly modified avian neck, with its reduced complex of muscles, has helped to allow birds to exhibit a variety of positions of their tracheae and esophagi. Skin might also act to restrict positioning. The extremely thin skin of a bird only serves to allow displacement, though the skin may still be regionally tight and constricting. Lastly, tracheal muscles of birds also affect tracheal positions. An interesting conundrum still exists though. That is, given the relative freedom given to the trachea and esophagus in birds we should expect to see a nearly fifty-fifty distribution of right-sidedness and left-sidedness, yet left-sidedness is an incredibly rare occurrence while right-sidedness is almost universal among birds. Another factor yet unknown must be affecting the sidedness creating a largely right sided preference. Given the ubiquity of tracheal and esophageal displacement within extant birds, extinct birds almost undoubtedly were characterized by displacement as well; certain fossils preserving tracheal and/or esophageal elements will be the subject of a later paper. The obvious question remains, as some crocodylians show displacement and birds unequivocally display tracheal and esophageal displacement, were dinosaurs characterized by this trait as well? The question will also be the subject of a future paper.

## Supporting Information

S1 Fig *Struthio camelus*. Live, captive individual. The gradual displacement of the trachea and esophagus to the right side is clearly seen.
(TIF)

S2 Fig Canada goose tracheal looping and the turkey. (A) *B*. *canadensis* tracheal elongation and looping. (B) *Meleagris gallopavo* displaying gradual tracheal and esophageal displacement.
(TIF)

S3 Fig Gruiformes and Cuculiformes. (A) *Fulica americana*, ventral view. (B) *F*. *americana* radiograph, lateral view. (C) *Rallus limicola*, lateral view. (D) *Coccyzus americanus*, ventral view. Tra = trachea; Eso = esophagus.
(TIF)

S4 Fig Extent of tracheal rotation in *Buteo platypterus*. The trachea is clearly seen to be twisted nearly 180˚ while coursing along the midline of the neck. Eso = esophagus.
(TIF)

S5 Fig Strigiformes. (A) *Megascops asio*. (B) *Strix varia*. (C) *Bubo virginianus*. (D) *B*. *virginianus* radiograph. Tra = trachea; Eso = esophagus.
(TIF)

S6 Fig Coraciiformes, Piciformes, Falconiformes, and Psittaciformes. (A) *Megaceryle alcyon*. (B) *Sphyrapicus varius*. (C) *Amazona ventralis*. (D) *Falco sparverius*.
(TIF)

S7 Fig Passeriformes. (A) *Hirundo rustica*. (B) *Wilsonia canadensis*. (C) *Cardinalis cardinalis*. (D) *Bombycilla cedrorum*. (E) *Seiurus aurocapilla*. (F) *Seiurus aurocapilla* ventral view. (G) *Turdus migratorius*. (H) *T*. *migratorius* oblique view showing the entrance of the trachea and esophagus into the thorax. Tra = trachea; Eso = esophagus.
(TIF)
