# Supplementary figures and images for: On the Morphological Description of Tracheal and Esophageal Displacement and Its Phylogenetic Distribution in Avialae
Source: PLoS One. 2016 Sep 20;11(9):e0163348. doi: 10.1371/journal.pone.0163348 (PMC5029910; doi:10.1371/journal.pone.0163348)

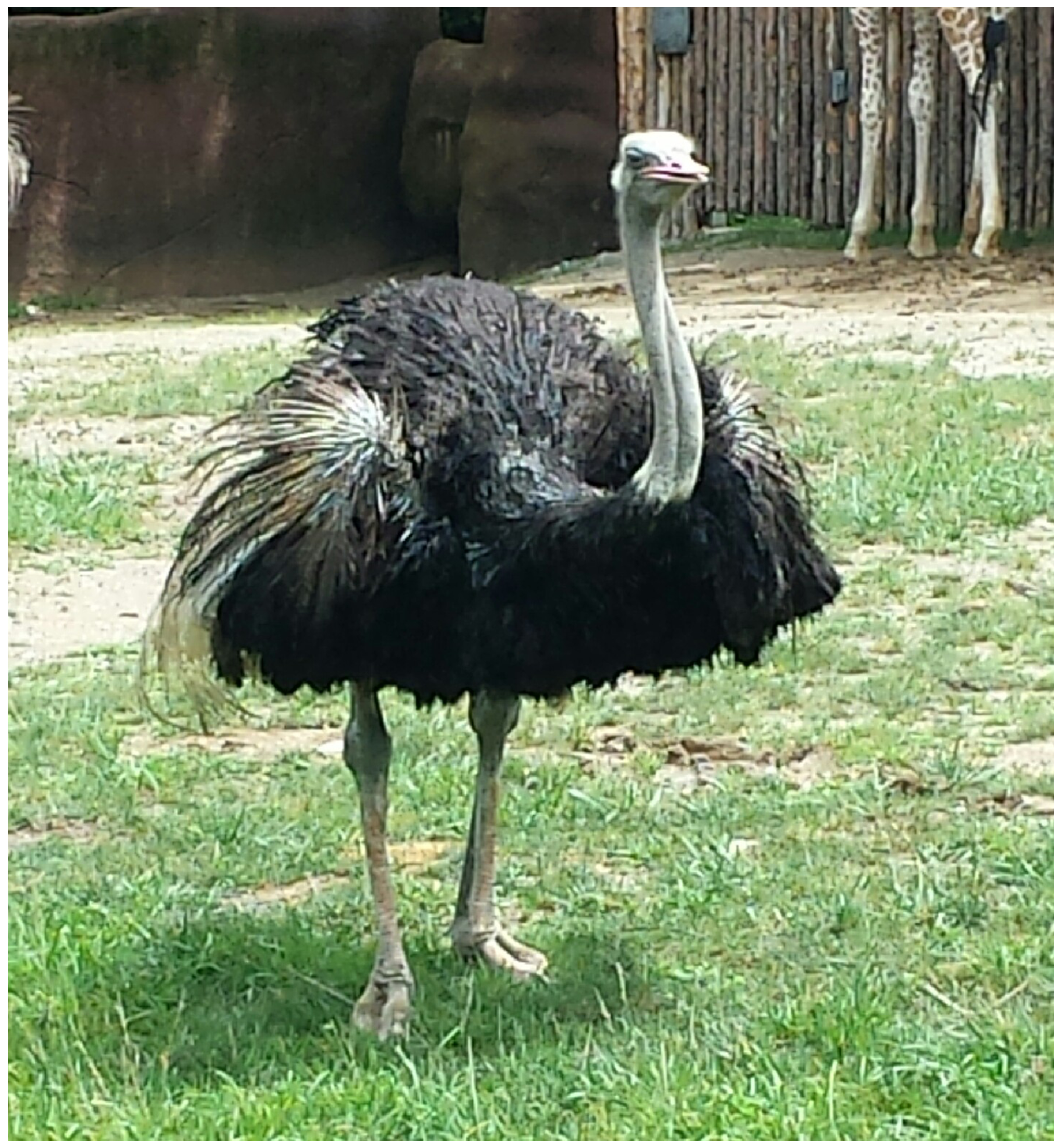

Supplement: S1 Fig — Live, captive individual. The gradual displacement of the trachea and esophagus to the right side is clearly seen. (TIF) [file pone.0163348.s001.tif]

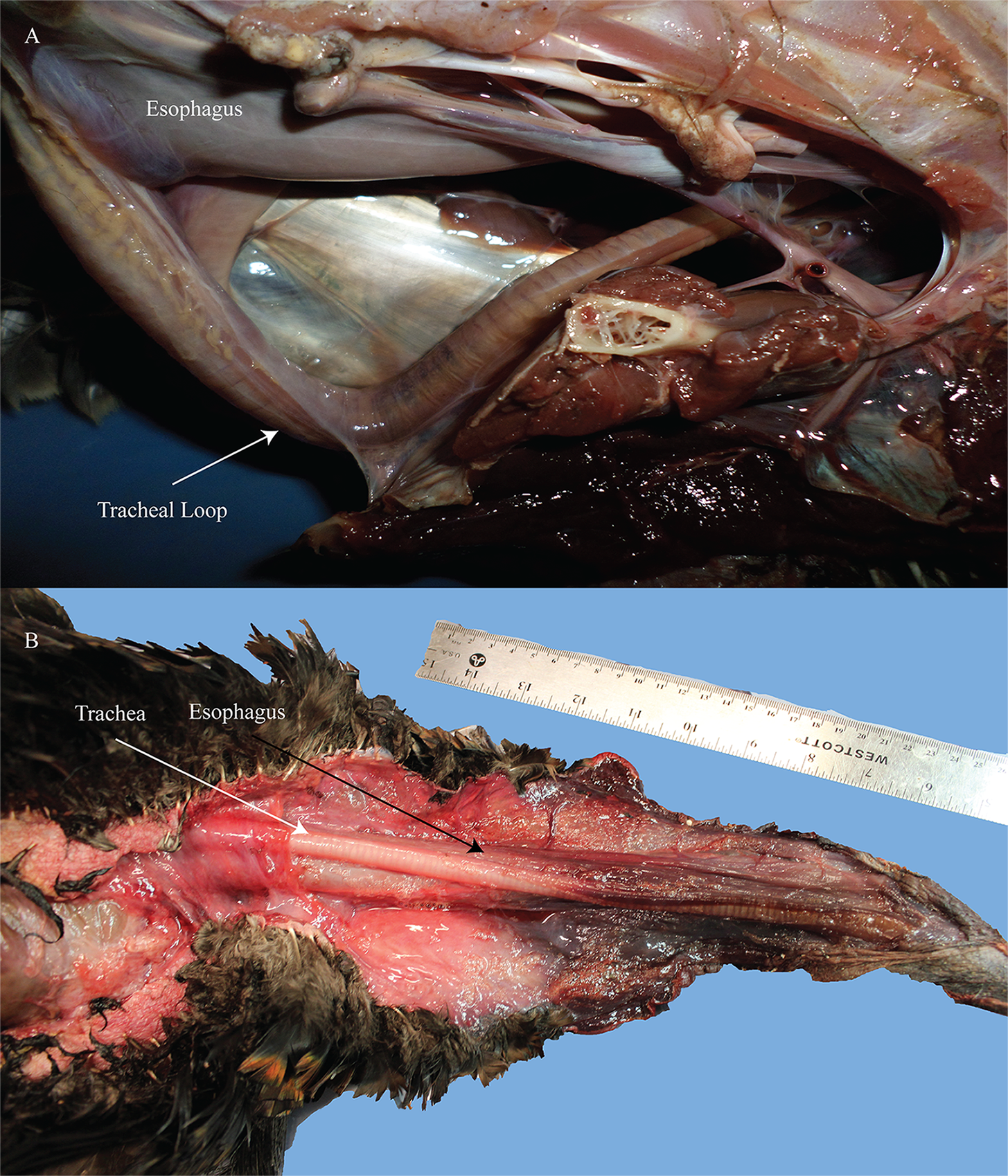

Supplement: S2 Fig — (A) B. canadensis tracheal elongation and looping. (B) Meleagris gallopavo displaying gradual tracheal and esophageal displacement. (TIF) [file pone.0163348.s002.tif]

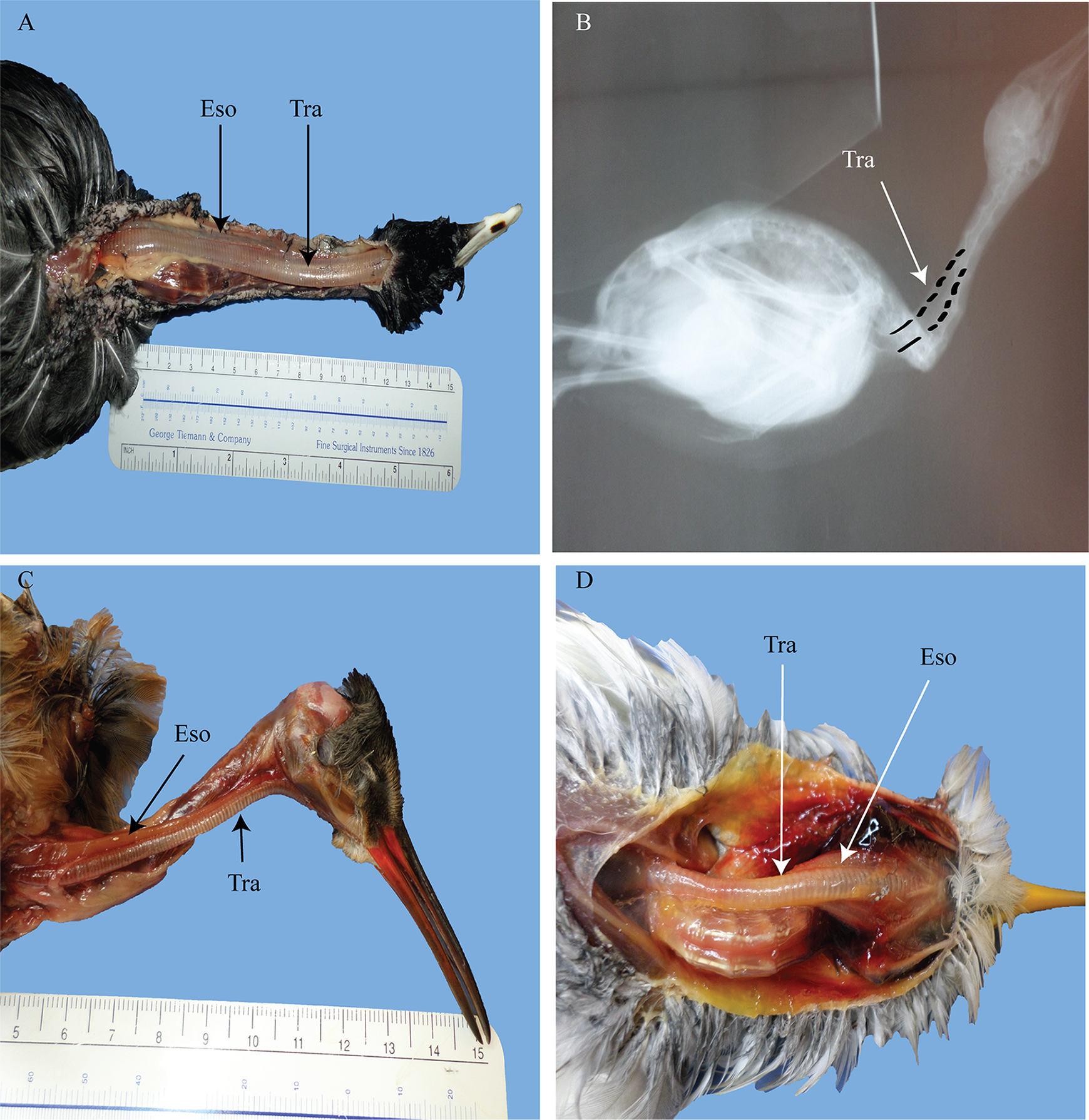

Supplement: S3 Fig — (A) Fulica americana, ventral view. (B) F. americana radiograph, lateral view. (C) Rallus limicola, lateral view. (D) Coccyzus americanus, ventral view. Tra = trachea; Eso = esophagus. (TIF) [file pone.0163348.s003.tif]

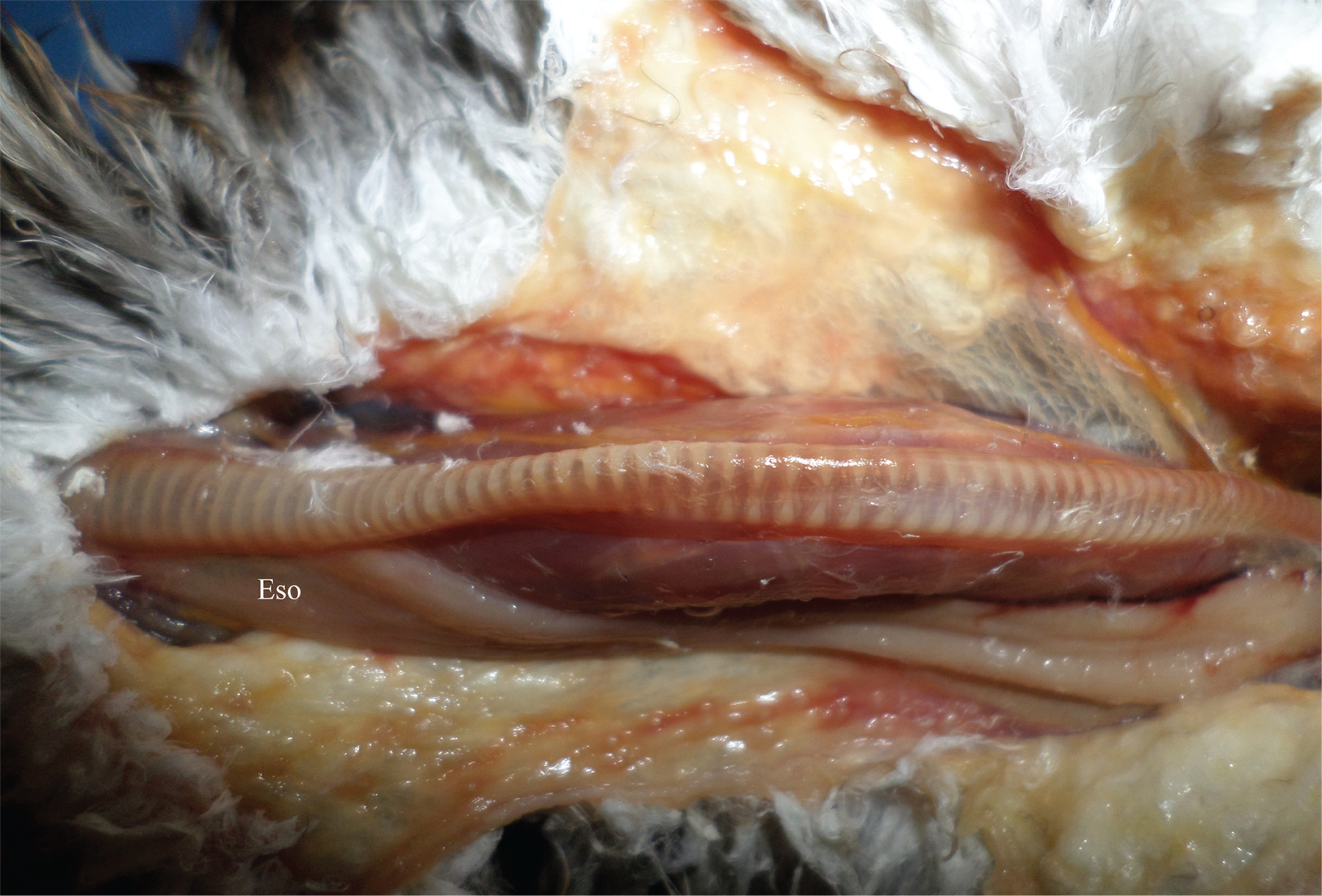

Supplement: S4 Fig — The trachea is clearly seen to be twisted nearly 180˚ while coursing along the midline of the neck. Eso = esophagus. (TIF) [file pone.0163348.s004.tif]

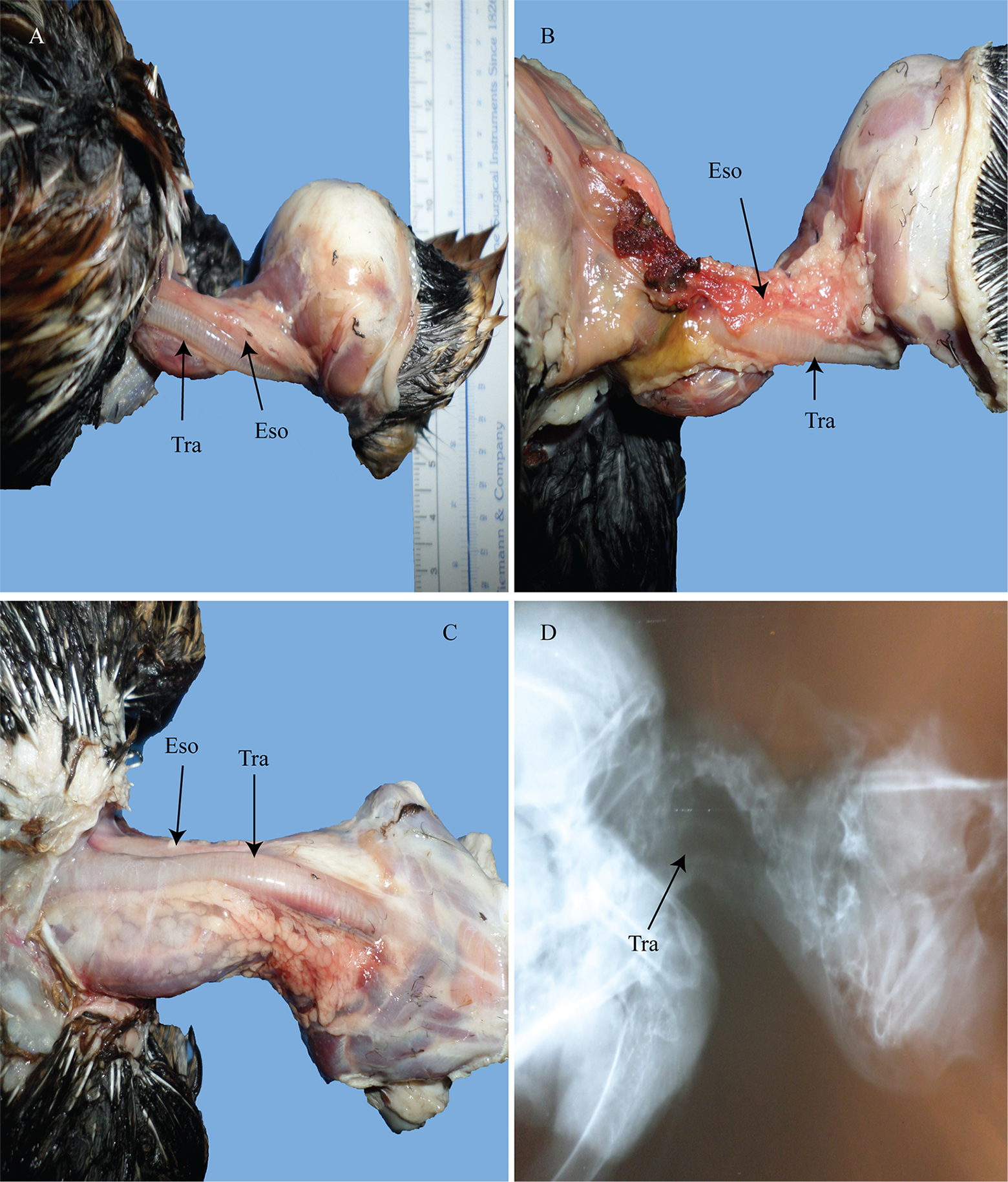

Supplement: S5 Fig — (A) Megascops asio. (B) Strix varia. (C) Bubo virginianus. (D) B. virginianus radiograph. Tra = trachea; Eso = esophagus. (TIF) [file pone.0163348.s005.tif]

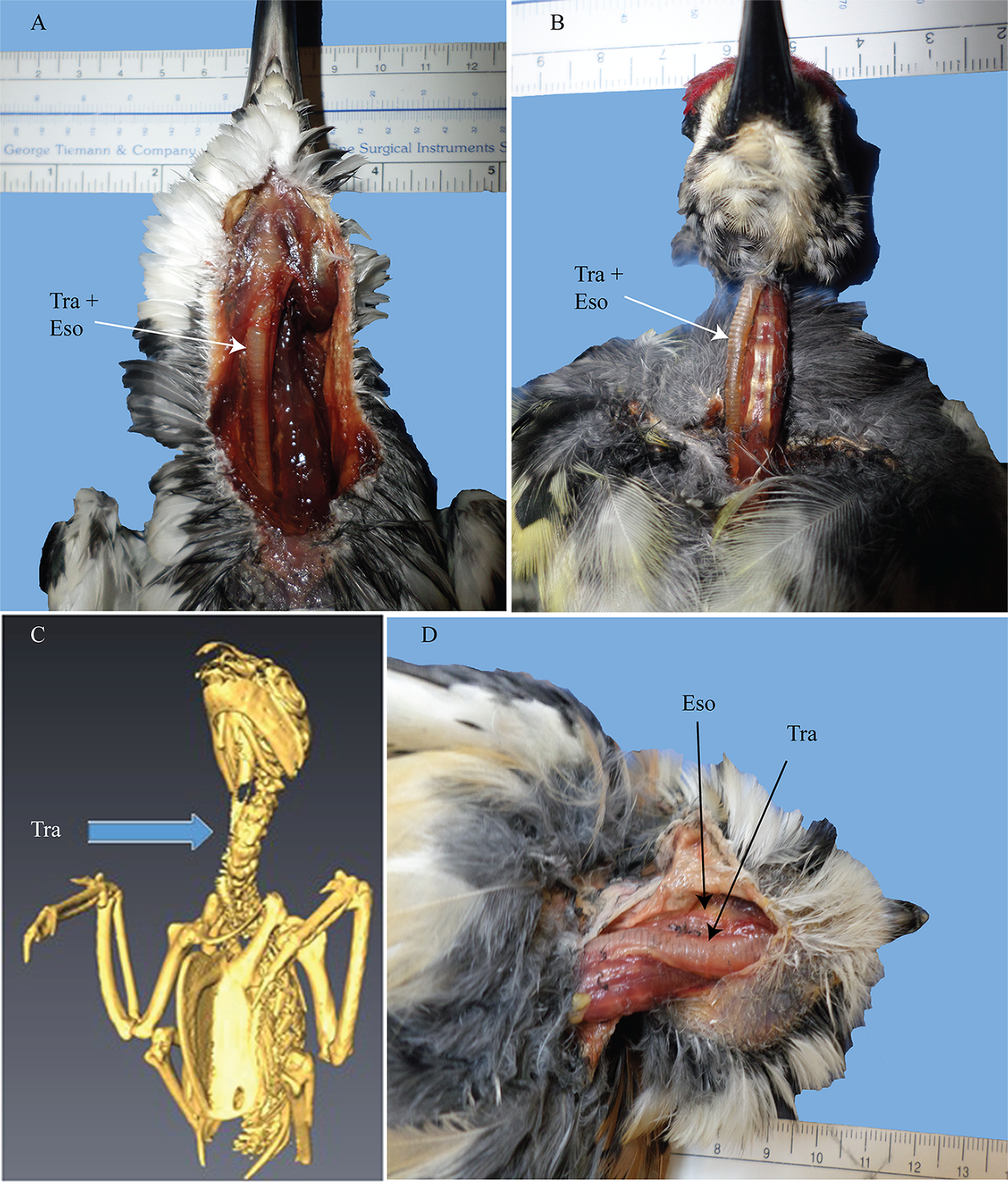

Supplement: S6 Fig — (A) Megaceryle alcyon. (B) Sphyrapicus varius. (C) Amazona ventralis. (D) Falco sparverius. (TIF) [file pone.0163348.s006.tif]

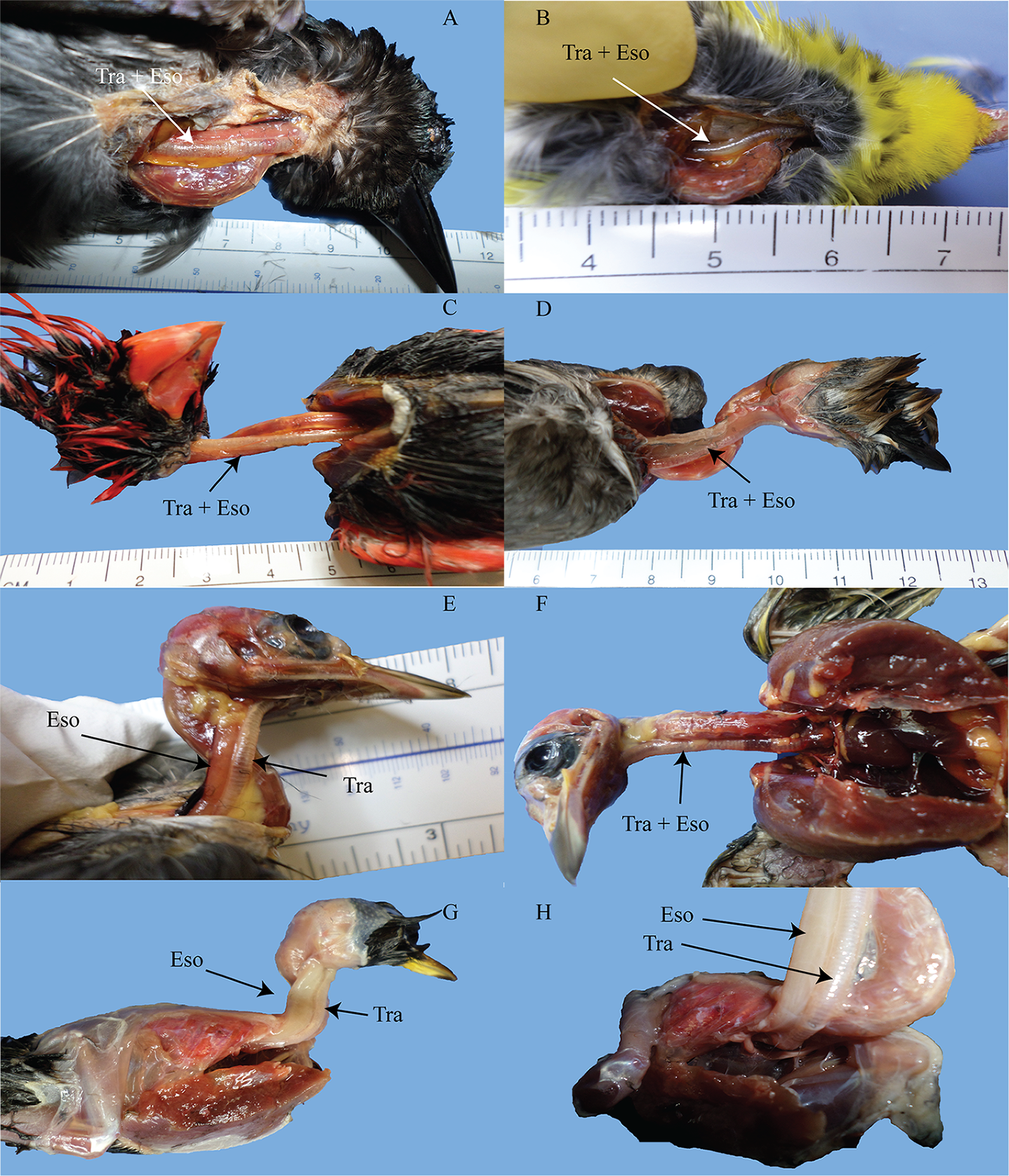

Supplement: S7 Fig — (A) Hirundo rustica. (B) Wilsonia canadensis. (C) Cardinalis cardinalis. (D) Bombycilla cedrorum. (E) Seiurus aurocapilla. (F) Seiurus aurocapilla ventral view. (G) Turdus migratorius. (H) T. migratorius oblique view showing the entrance of the trachea and esophagus into the thorax. Tra = trachea; Eso = esophagus. (TIF) [file pone.0163348.s007.tif]
